# Winter is (not) coming: Warming temperatures will affect the overwinter behavior and survival of blue crab

**DOI:** 10.1371/journal.pone.0219555

**Published:** 2019-07-25

**Authors:** Hillary Lane Glandon, K. Halimeda Kilbourne, Thomas J. Miller

**Affiliations:** Chesapeake Biological Laboratory, University of Maryland Center for Environmental Science, Solomons, Maryland, United States of America; Universidade de Aveiro, PORTUGAL

## Abstract

Understanding how increases in water temperature may affect winter dormancy period duration and overwinter survival are important for the effective conservation and management of estuarine species in the face of a warming climate. In this study, we determined the length of the overwintering period and the probability of overwinter survival of blue crab (*Callinectes sapidus*), an ecologically and economically important estuarine crustacean. Overwintering period length and probability of overwinter survival were determined using projected water temperatures up to the year 2100, derived from a harmonic model that utilized air temperatures from multi-model ensemble of regional-scale climate projections. Our estimates of warming water temperatures by 2100 in Chesapeake Bay indicate that winters will be up to 50% shorter and overwinter survival will increase by at least 20% compared to current conditions. The warmer conditions will lead to faster and prolonged seasonal growth, which, when combined with lower winter mortality, will lead to increased population productivity. The level of expression of this increased productivity will depend on the response of other elements of the Chesapeake Bay food web, as well as possible changes to fishery management policies over the same time period.

## Introduction

Dormancy is a life history strategy employed by a variety of species to survive periods of environmental hardship and can be initiated by external cues or by an internal physiological response [1, 2]. Environmental variability within a species’ range may cause interannual variability in the proportion of the population undergoing dormancy and overwinter survival rates [3, 4]. The blue crab, *Callinectes sapidus*, is an ecologically and economically valuable marine crustacean that is found in coastal areas of the western Atlantic and the Caribbean from Argentina to New England [5–7]. Like all crustaceans, blue crab grow by molting, which involves rapid increases in size associated with the shedding of the external carapace (growth per molt) followed by periods of stasis (intermolt period). Previous research has shown that the intermolt period, but not growth per molt, is temperature-dependent [8, 9]. Therefore, growth rate and consequently key life history events such as maturation, are determined by the external temperature [8]. The critical temperature for growth in blue crab is approximately 9°C, below which metabolic rates decline and dormancy is induced, referred to as overwintering in this species [8, 10]. As a result of these specific temperature-dependencies, the latitudinal temperature gradient present throughout the blue crab range creates predictable variability in stage duration, growth rates and the likelihood of winter dormancy [11, 12]. Moreover, the intensity and duration of overwintering behavior has been directly linked the winter survival of blue crab [3] and therefore changes to overwintering behavior may affect blue crab population dynamics and geographic distribution. Understanding how current warming trends will affect the survival and life history of marine species is critical to effectively managing these ecosystems in the face of a changing climate.

Anthropogenic release of fossil fuels has caused an increase in the concentration of carbon dioxide (CO_2_) in the atmosphere from pre-Industrial Revolution levels of approximately 280 ppm [13]. Since 1990, the Intergovermental Panel on Climate Change (IPCC) has been using models of climate processes to predict future changes in climate on a global scale [14]. Global Climate Models (GCMs) incorporate appropriate fluid dynamics of the ocean and atmosphere with other climate system processes including the carbon cycle, dynamic vegetation, atmospheric chemistry and land ice. Atmospheric CO_2_ is expected to continue increasing through the end of the 21^st^ century due to the continued burning of fossil fuels [14–16]. Due to the greenhouse effect, this increase in CO_2_ has and will continue to cause an increase in atmospheric and, consequently, ocean temperatures, which are projected to warm by 2.6–4.8°C by the year 2100 [14] under the Representative Concentration Pathway 8.5 (RCP8.5) greenhouse gas scenario. Considering the ecological and economic value of nearshore and coastal ecosystems [17, 18], determining the effects of predicted increases in global temperatures on marine species life histories in these systems is important to the understanding of the future of these critically important systems. Such questions regarding the effects of climate change require forecasts at regional, rather than global scales. Downscaling GCM projections to higher spatial resolution in specific regions addresses this issue by helping us understand how global change may play out in specific environments [19, 20].

**Fig 1:**
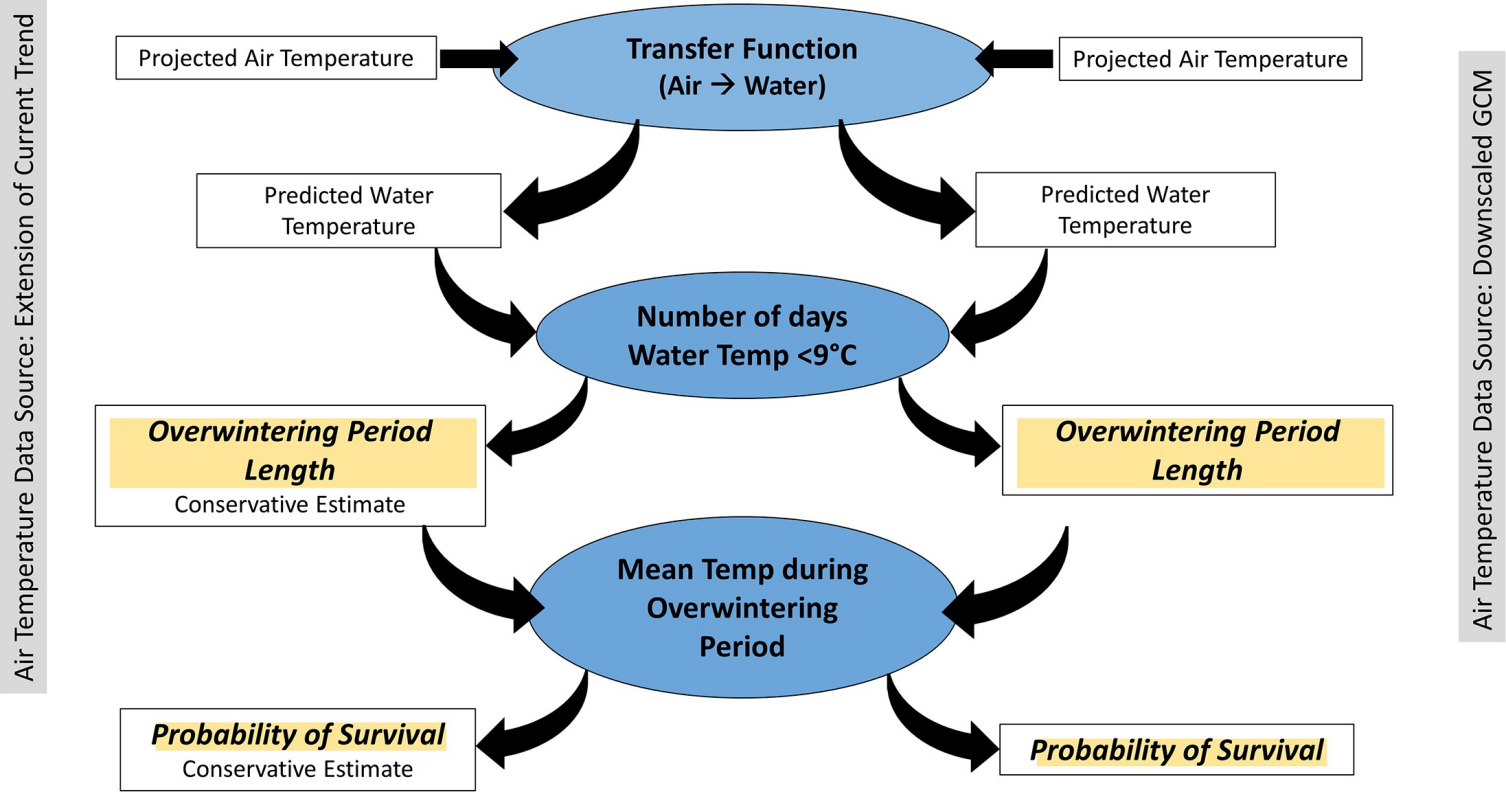
Schematic of the utilization of the transfer function to predict the length of the overwintering period and the winter survival of blue crab through the year 2100. Daily projected air temperature was accessed from two sources: a conservative extension of the observational trend of increasing water temperature in the historical data (left side of figure) and a multi-model ensemble of downscaled CGM projections (right side of figure). The projected air temperature time series were used with the transfer function to predict future water temperature based on those two data sources. The number of days below 9°C for each source of future temperature data was used as a proxy for the overwintering period of crabs under both the conservative (extension of the observational data trend) and the downscaled GCM warming scenarios. The mean temperature during the overwintering period was used in conjunction with the length of the overwintering period to predict the probability of overwinter survival for each warming scenario.

The goal of this study was to use a multi-model ensemble of regional, downscaled GCM temperature projections to explore how changes in water temperature may impact the overwintering behavior and winter survival of blue crab in the Chesapeake Bay. To achieve this goal, we accessed a long-term (1938–2016) dataset of daily water and air temperature measurements collected in the Patuxent River. These temperature data were fit to a harmonic regression model that yielded a transfer function which was used to estimate water temperature from air temperature [21]. This transfer function was then applied to two sources of climate projections, chosen to characterize a conservative estimate represented by an extension of the temperature trend in the long-term dataset, and downscaled climate model-based estimates of a relatively high greenhouse gas emissions future (RCP8.5[22, 23]) to predict water temperatures in the Patuxent River out to 2100 C.E. (Fig 1, details in methods).

## Methods

### Observational data collection and processing

Air and water temperatures have been observed since 1938 at the end of a 274 meter long pier located at the Chesapeake Biological Laboratory (CBL), Solomons, MD (38.3185° N, 76.4541° W). Observations were taken manually at noon using a research-grade mercury thermometer from 1938–2012. From 2012 to 2016, water temperatures were recorded every 15 minutes via a calibrated YSI EX02 multi-parameter sonde (Yellow Springs Instruments, Inc., Yellow Springs, OH)., and air temperature data were recorded every 15 minutes via Davis Vantage Pro 2 (model 6152) meteorological station (Davis Instruments, Haywood, CA). Although this 72-year time series is largely complete, missing values were replaced with nearby (co-located or <2 nautical miles) observational data from the Chesapeake Bay Program (CBP) and the National Oceanic and Atmospheric Administration (NOAA) buoy system. Missing values in CBL air temperature data were filled in using data from NOAA buoys at Cove Point (buoy number 8577018, COVM2) and Solomons (buoy number 8577330, SLIM2) and missing values in CBL water temperature were filled in using data from CBP water quality stations LE1.3 and LE1.4 and NOAA Solomons buoy (buoy number 8577330, SLIM2). Major axis regression of the CBL data with all other sources indicated a high degree of similarity between data sources (Slope>0.94, R^2^>0.95, *P*<0.01 for all regressions). Gaps in the combined historical sea surface temperature time series, primarily due to no data collection over weekends and holidays, were filled in by linear interpolation because water temperatures do not often change substantially over the course of a few days due to the high specific heat of water. This was tested by an autocorrelation analysis of the daily data, which demonstrated a temporal decorrelation scale of 91 days. The average gap length in the air temperature time series was 10 days and the longest gap was 20 days. The average gap length in the water temperature time series was 9 days and the longest gap was 17 days. The filled gaps represented 15.6% and 12.4% of the total CBL air and water temperature time series, respectively. The observational data used in this study are available in S1 Table.

### Estimation of the harmonic model and transfer function

Harmonic models were developed to characterize the seasonal cycle of air and water temperature known to exist in mid-latitude estuaries following the methods of Cho and Lee [21]. Full details are provided in Glandon [24] and are only summarized here. The multistep approach includes both calibration (C) and validation (V) phases. In the calibration phase, 30% of the observations, drawn at random from the entire dataset, are used to fit harmonic models to express air temperature and water temperature as a function of Julian day. The parameter estimates from the air and water calibration temperature models are subsequently validated using the 70% of the data not included in the original model fit. The parameters from the validated models are then combined in a transfer function that predicts water temperature from air temperature. A final model is developed to predict future water temperature using new linear and harmonic coefficients based on the entire observational dataset. The performance of the final model is evaluated by comparing the root mean squared error (RMSE) of the validation model to the RMSE of the final model.

The transfer function was given by the following Eq (1):

$${WT}_{E}(t_{i})=B_{0}+B_{1}t_{i}+\sum \left[cr_{m}*{CA}^{V}*\cos\left(w*t_{i}\right)+sr_{m}*{SA}^{V}*\sin\left(w*t_{i}\right)\right]$$ (1)

where WT refers to expected water temperature, t_i_ is time (day of year), *B*_*O*_ is the intercept, and *B*_*1*_ is the slope of the relationship used to characterize the change in daily temperature over the reference period. *CA* and *SA* are the harmonic cosine and sine coefficients, and *cr*_*m*_ and *sr*_*m*_ are ratios of the parameter estimates from similar models fitting either water temperature or air temperature individually, i.e., cr_m_ = $CW_{m}^{C}/CA_{m}^{C}$ and sr_m_ = $SW_{m}^{C}/SA_{m}^{C}$. The superscripts C and V denote the calibration and validation model stages, respectively and the frequency term is expressed as *w* = 2π/365.25.

### Projection of future water temperature

The transfer function (Eq 1) was used to predict daily future water temperature under two assumptions regarding future climate: extension of the trend in observational air temperature data (extended data) and a multi-model ensemble of downscaled GCM air temperature data (downscaled GCM) from model runs forced with Representative Concentration Pathway 8.5 (RCP8.5). The least squares parameter estimates of the transfer function were used directly to extend patterns in the observed data from 2016–2100. The downscaled GCM data [25] consisted of 41 ensemble members from 20 models provided by 15 modeling groups from the Climate Model Intercomparison Project, CMIP5 (S2 Table). The data were downscaled using a bias correction constructed analog technique (BCCAv2; [26]) and downloaded from the CMIP3 and CMIP5 downscaled climate and hydrology projections archive at https://gdo-dcp.ucllnl.org/downscaled_cmip_projections/. Daily minimum and maximum 1/8 degree resolution data from 3 grid boxes along the US east coast near Solomons were downloaded for January 1950-December 2099 and averaged to create a single daily observation of air temperature.

Uncertainties in forecasted temperatures were calculated differently for the extended data and the downscaled GCM projections. For the extended data, we resampled regression parameters from a normal distribution defined by the mean and standard deviation (SD) of the original parameter estimate, generating 50 new forecasted temperature projections. This created 50 separate projected temperature time series, and we subsequently calculated the daily mean (± SD) temperature of the projected time series. For the downscaled GCM projections, we carried forward the projections of air temperature from each of the 41 individual ensemble members and then calculated mean (±SD) daily temperature.

### Determination of changes in overwintering period of crab

The total number of days when water temperature was projected to be below the critical temperature for growth, (9°C; [8]) was determined for the two sources of time series of projected water temperature (extended data and downscaled GCM). The number of days below 9°C represents the length of the overwintering period of crab in any given year and is defined as the length of winter in this study. Winter years were determined as the bridge of calendar years (e.g.: winter 1990–1991 was recorded as winter 1990) since wintertime temperatures occur between calendar years in the Chesapeake Bay. Due to the increase in year length during leap years, the length of winter was often one day longer than would be expected during these years. This is evident in the oscillations present in the predicted length of winter and overwinter survival (see Results), and represent a calculated increase in winter length and concurrent decrease in overwinter survival during those years, despite the stability of the actual orbital parameters controlling winter.

Changes in the length of the overwintering period with year were examined using linear regression. The length of the overwintering period predicted by the extended data was compared to the length of the overwintering period predicted by the downscaled GCM data in four time intervals (2017–2030, 2031–2050, 2051–2070, 2071–2100) using a t-test.

### Probability of overwinter survival of crab through the year 2100

Overwinter survival through the year 2100 was determined using the length and severity of winter. The length of winter for each year was determined as described above. Winter severity was quantified as the average temperature during the overwintering period. Based upon the findings of Bauer and Miller [3], the following Eq (2) was used to determine the probability of overwinter survival:

$$S(t)=exp(-\lambda t^{\lambda }*exp[-\lambda *(3.59+0.10*(Temp)+0.02*(Sal)+0.03*(Size)]$$ (2)

where t is the overwinter duration in days, λ is a rate parameter (1/scale). Temperature (Temp) was the average temperature during the overwinter period for a given year based upon the analysis conducted above, salinity (Sal) was the average salinity in the mid Chesapeake Bay from 2010–2016 [27], and crab size (Size) was chosen to represent the juvenile life stage (40mm carapace width). Changes in the probability of overwinter survival with year were examined using linear regression. The probability of overwinter survival based upon the extended data was compared to the probability of overwinter survival based upon the downscaled GCM data in four year groups (2017–2030, 2031–2050, 2051–2070, 2071–2100) using a t-test.

Data processing and analyses were conducted in R (version 3.2.2; [28]) using R-Studio (version 0.98.1103) using an alpha level of P < 0.05 for all analyses. The full model code is available in S1 Appendix.

## Results

**Fig 2:**
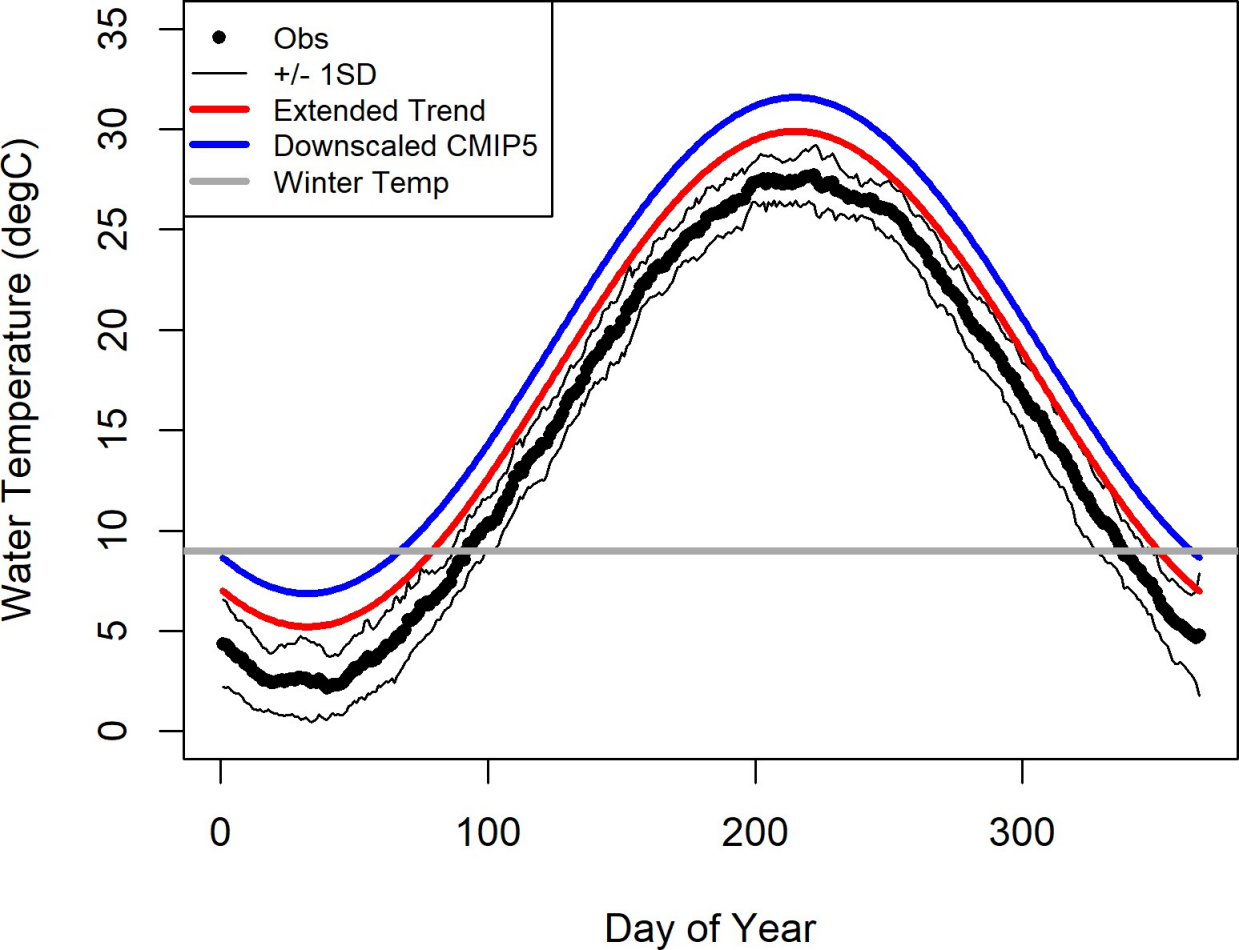
**Climatology of observed historical water temperature (Obs) in the reference period (1961–1990) and the projected future water temperature (red and blue lines) in the prediction period (2070–2099).** The grey line is at 9°C and represents the temperature at which crabs will commence overwintering. Both the conservative estimate of the increase in water temperature (Extended Trend, red line) and the projected water temperature from the multi-model enseble of downscaled GCM models (Downscaled GCM, blue line) predict the average year in the prediction period will be warmer than the average year in the reference period.

**Fig 3:**
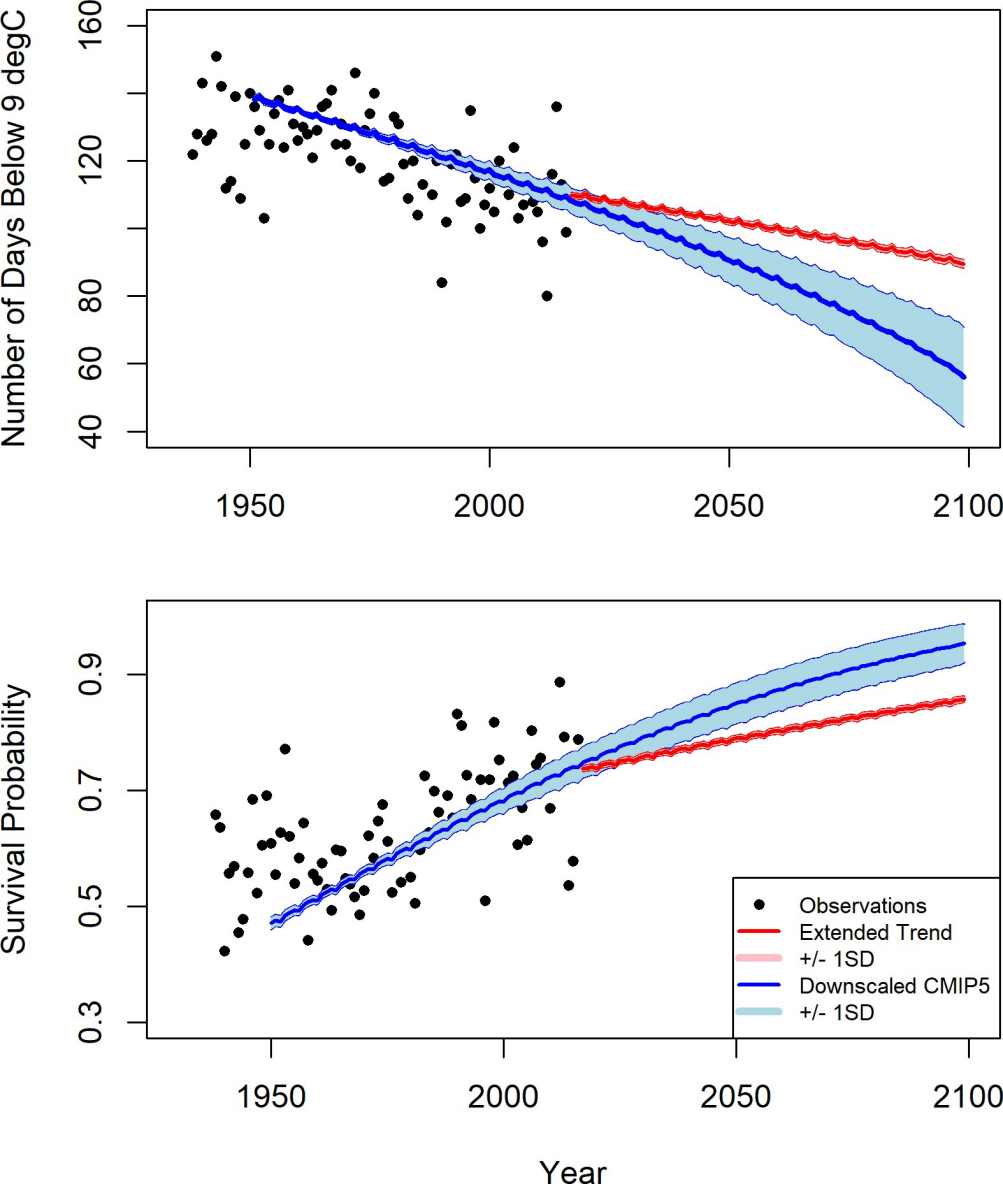
**Length of crab overwintering period (top panel; expressed as the number of days below 9°C in a given year) and probability of overwinter survival (bottom panel) for observational data (black dots) and projected data from two sources (conservative estimate (Extended Trend) in red and a multi-model ensemble of downscaled GCM data (Downscaled GCM) in blue).** The year 1977 was excluded from the analysis due to large gaps in the observational data for that year. Error bands for projections represent one standard deviation.

Our analyses indicate that under the tested scenarios, future (2070–2100) water temperature in the Maryland portion of the Chesapeake Bay will increase from the 1961–1990 average of 15.2 ±8.9°C (standard deviation) to 17.6 ± 8.6°C extending the current trend, and to 19.2±8.6°C using the downscaled-models multi-model ensemble average. The temperature seasonality of an average year in the reference interval (1961–1990) compared to an average year in the prediction interval (2070–2099) under both future temperature scenarios is shown in Fig 2. The length of the overwintering period in the long-term observational data ranged from 145 days in 1943 to 75 days in 2012, with an average length of 117±16 days. Using an empirically determined, temperature dependent survival function [3], the probability of overwinter survival in the observational data ranged from 0.44 in 1940 to 0.95 in 2013 with an average probability of overwinter survival of 0.65±0.11. Despite high interannual variability, a significant decline in the length of the overwintering period with time was observed in the long-term observational data (*P*<0.05, R^2^ = 0.37; black dots in Fig 3 –top panel). Similarly, there was a significant increase in the probability of overwinter survival in the long-term observational data (*P*<0.05, R^2^ = 0.26; black dots in Fig 3 –bottom panel).

The conservative approach projected a decline in the length of the overwintering period to 90±1 days and an increase in the probability of overwinter survival to 0.86±0.01 by the year 2100 (red lines in Fig 3 - top panel). The multi-model ensemble of downscaled climate model data projected a decline in the length of the overwintering period to 56±15 days and an increase in the probability of overwinter survival to 0.95±0.03 by the year 2100 (blue lines in Fig 3 –top panel). A t-test indicated significant differences in the estimated length of the overwintering period and the probability of overwinter survival between the conservative and downscaled approaches (2017–2030, 2031–2050, 2051–2070, 2071–2100; P<0.05 for all year groups for both tests). As the uncertainties in our estimates of overwintering period length and overwinter survival are based on long-term trends and the interannual variability in these projections is expected to be much larger than the uncertainty bounds quantified here.

## Discussion

Our analysis of historical and future predicted temperatures indicates that water temperatures will continue to rise in the Chesapeake Bay through the year 2100. This increase in water temperature will occur equally in all seasons of the year, and will therefore effect blue crab wintertime behavior and survival. The projected increases in water temperature will result in a significant shortening of the overwintering period of blue crab in the Chesapeake Bay. We predict that the shortening of winter combined with increases in average wintertime temperatures will cause a significant increase in blue crab winter survival. The concurrent decline in winter length and increase in winter survival has the potential to significantly change the biology of blue crab in the Chesapeake Bay.

**Fig 4:**
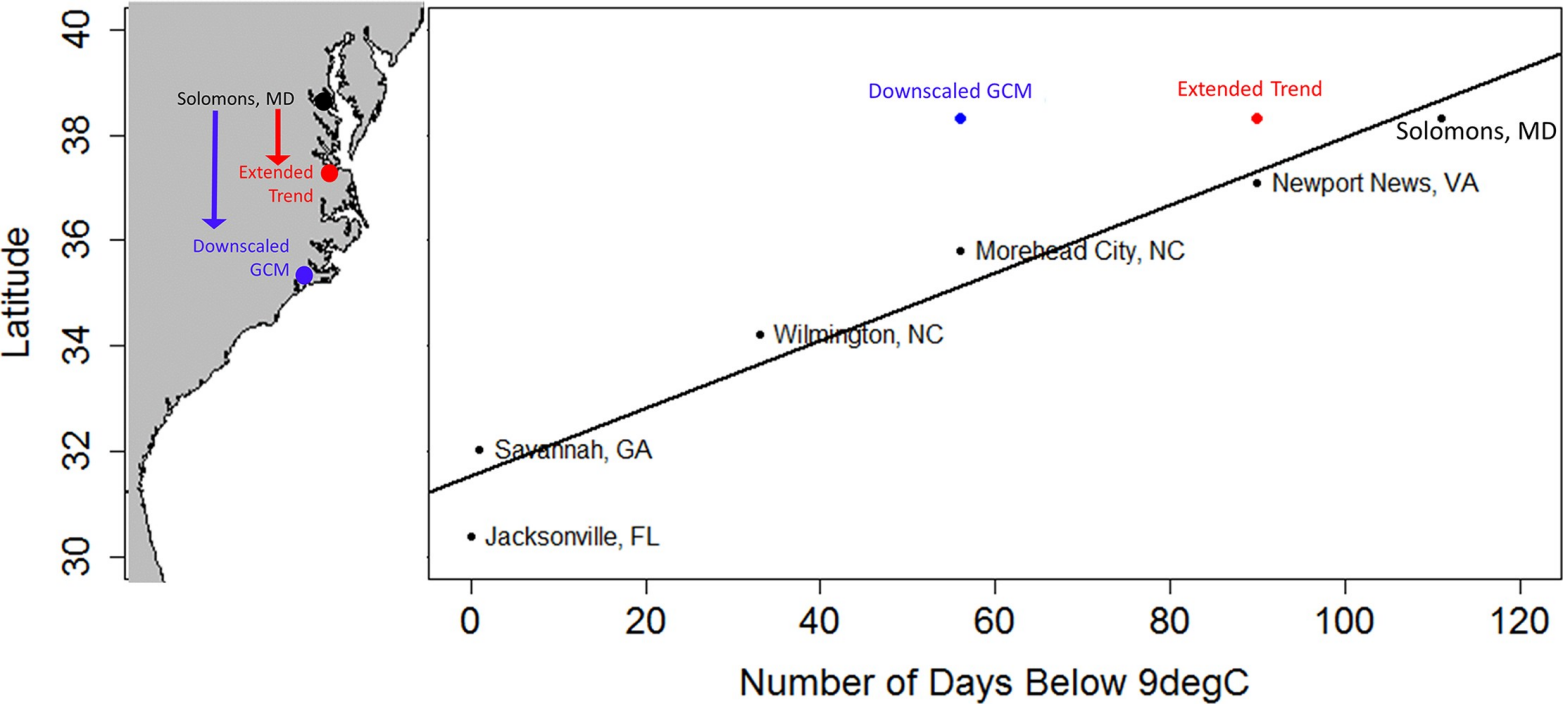
Number of days below 9°C by latitude at different locations along the east coast of the US from 2012–2016. Points in black indicate observational data from the CBL Pier and the National Data Buoy Center. The black line is the linear regression of the observations with latitude. The red and blue points indicate the location equivalent to number of days below 9°C projected by the extended trend and the multi-model ensemble of downscaled GCM data, respectively. Direct extension of trend in the observational data predicted a shortening of the overwintering period from 117±15 days to 90±1 days and the downscaled GCM model data predicted a shortening of the current overwintering period to 56±15 days.

To assess the potential impacts of the projected changes in wintertime length and severity on the Chesapeake Bay crab population, we compared present-day overwintering behavior in the Chesapeake Bay with crab populations from latitudes that are currently characterized by the projected future conditions for the Bay. The number of days below 9°C at various latitudes along the east coast of North America from 2012–2016 were estimated from the NOAA National Buoy Data Center and are shown in Fig 4. According to a regression of the number of days below 9°C and latitude, the number of days below 9°C in the year 2100 projected by the extended data is equivalent to the current number of days below 9°C in Newport News, Virginia and the number of days below 9°C in the year 2100 projected by the downscaled model data is equivalent to the current number of days below 9°C in Morehead City, North Carolina. Since the overwintering behavior of blue crab is a response to a seasonal decrease in environmental temperature [29], it is reasonable to assume that the future overwintering behavior of blue crab in Chesapeake Bay will change to resemble the behavior currently observed in the Sounds of North Carolina and further south as temperatures continue to warm. Assuming similar variability in the length of winter in the future as was observed in the past, the probability of crab overwintering in Maryland will decline and in some years individual crabs in this region may not overwinter at all. We infer from our analyses that blue crab overwintering behavior in the Maryland portion of the Chesapeake Bay will change from a pattern in which blue crab overwinter consistently to one in which overwintering of blue crab occurs inconsistently, sensitive to winter severity.

Our analysis indicates that not only will blue crab overwintering behavior become more unpredictable, but also the probability of overwinter survival will increase by at least 20% by the year 2100, nearing 100% overwinter survival under RCP8.5 by the end of the century. These changes will bring about an increase in the length of the growing season, and a higher level of population productivity, with potential consequences to the management of blue crab in Chesapeake Bay [30]. Currently, crabbing is prohibited during the overwintering period (December-March) in all three management jurisdictions in the Chesapeake Bay. This creates a *de facto* closed season for crab during the winter. The positive response of the population to the closure of a winter fishing in the lower Chesapeake Bay in 2008 shows the role of this closed season in maintaining the Chesapeake Bay crab population at sustainable levels [31]. However, the increase in temperature and subsequent increase in wintertime crab activity may encourage a lengthening of crabbing season, as is currently the practice in the lower latitude states where crabs are active year-round. Although it is possible that the increases in growth rate and survival resulting from increased temperature could sustain a longer crab fishery, the interannual variability in overwintering behavior in the Maryland portion of the Chesapeake Bay in the future complicates the wintertime management of this species. Reductions to, or elimination of, the wintertime closure in response to increases in average wintertime activity might drastically reduce population levels during cold years when crab will likely still overwinter. Therefore, despite the warming trends observed in this study, until crabs are consistently active year-round, the elimination of the wintertime dredge fishery closure may put immobile, overwintering individuals at greater risk of mortality during cold years.

The projected increases in temperature will not occur without other environmental changes resultant from climate change, including increases in atmospheric, and therefore oceanic *p*CO_2_. Although blue crab growth and oxygen consumption rates are not expected to be affected directly by increasing *p*CO_2_ [9, 32, 33], bivalve prey of crab are expected to experience decreased calcification and growth rates as water acidifies in the future [34]. The increase in energetic demands as a result of the predicted increase in the growing season combined with an increase in the vulnerability of crab prey in the more acidic water of the future may cause crab to target weaker prey items more heavily and could have ecosystem-wide effects. There is also evidence that the increased growth rates associated with increased temperature may come at a cost to blue crab carapace integrity [35]. In order to sustain increased growth rates when exposed to high temperature, the percent of high-magnesium calcite in the carapace declines, which has been associated with declines in carapace strength in other invertebrates [36–38]. Precipitation levels in the Chesapeake Bay are predicted to increase in the future, in addition to the predicted increases in *p*CO_2_ in this system [39]. Salinity is known to have a negative effect on survival and growth in blue crab [40, 41], which may counteract the increased growth expected as temperatures warm in this system in the future. The tradeoffs between the effects changes in temperature, acidity, and salinity underscore just some of the complexities involved in predicting the population level impacts of climate change, even on a single, well-studied species such as the blue crab.

Our analyses focused on the juvenile life stage of blue crab due to its importance in regulating overall population dynamics in the Chesapeake Bay [30]. However, crab size has also been shown to be a significant driver of winter survival rates [4]. Under current conditions, larger crabs have a significant survival advantage in the wintertime over their smaller counterparts. However, this advantage may not be as impactful as winter temperatures, and therefore crab survival rates across crab sizes, continue to rise, and could be manifested as a decline in average size at maturity, which has been observed in previous studies on blue crab [42]. Additionally, increased temperatures have been associated with declines in fecundity in blue crab [42] as well as changes to the timing of spawning and consequently larval distributions, with implications of mismatched food resources for early hatching larvae. The tradeoff between potential increases in maturation rates and possible declines in fecundity or mismatches in larval food availability are critical to understanding the overall effect of climate change on this species. From a fisheries perspective, understanding how our predictions at the juvenile life stage would play out throughout the ontogeny of blue crab in this system is also an important area of future research.

Considering the high environmental variability present in the estuarine system [43, 44], our analyses underscore the importance of adaptive management in order to mitigate the effects of increasing temperatures on commercially important species. Despite the significant decrease overwintering period length and a significant increase in overwinter survival of blue crab under both the conservative and extreme warming scenarios tested in this study, the variability in the projections creates uncertainty around blue crab overwinter behavior and survival. Although estuarine species such as blue crab are adapted to live in variable environments, the effect of increases in environmental variability on blue crab physiology are not yet know. Additionally, environmental parameters will likely not respond to climate changes at equal rates–*p*CO_2_ is expected to change much faster than temperature [45]–and the physiological response of species to these differing environmental parameters may be complex. The burden rests upon timely availability and interpretation of biological and environmental data in order to sustainably manage blue crab both ecologically and economically as temperatures and climate variability continue to increase through the next century.

## Supporting information

S1 Table Observational air and water temperatures used in this study. Columns are as follows: Date—take temperature reading was taken. CBL Water Temp (degC)—water temperature in degrees Celsius taken at CBL pier. Temperature was taken once daily using a mercury thermometer from 1938–2012. Temperature was taken every 15 minutes using a YSI (details in methods section) from 2012–2016. Temperature readings taken during this later time period were averaged per day. CBL Air Temp (degC)—same as CBL Water Temp but for air temperature. CBP Water Temp (degC)—water temperature in degrees Celsius taken by the Chesapeake Bay Program at stations LE1.3 and LE1.4 (details in methods section). NOAA Solomons Air Temp (degC)—air temperature in degrees Celsius at NOAA buoy number 8577330. NOAA Solomons Water Temp (degC)—same as NOAA Solomons Air Temp but for water temperature. NOAA CovePt Air Temp (degC)—air temperature in degrees Celsius at NOAA buoy number 8577018. NOAA CovePt Water Temp (degC)—same as NOAA CovePt Air Temp but for water temperature. Combined Air Temp (degC)—air temperature for all data sources combined. If an observation was recorded at CBL, that value was used first. Then the data sources were used in the following order: NOAA Solomons, CBP, CovePt. This created the most complete observational data set possible. Combined Water Temp (degC)—same as Combined Air Temp but for water temperature.
(XLSX)

S2 Table Research groups that contributed model ensembles to the downscaled GCM data used in this study. Climate model data were downloaded from the CMIP3 and CMIP5 downscaled climate and hydrology projections archive at https://gdo-dcp.ucllnl.org/downscaled_cmip_projections/. See methods section of the manuscript for details on the specific parameters used to access the data from these research groups.
(DOCX)

S1 Appendix R code for harmonic model, future temperature predictions, and future crab survival. (DOCX)
