# Supplementary material for: Winter is (not) coming: Warming temperatures will affect the overwinter behavior and survival of blue crab
Source: PLoS One. 2019 Jul 25;14(7):e0219555. doi: 10.1371/journal.pone.0219555 (PMC6657847; doi:10.1371/journal.pone.0219555)
Supplement: S2 Table — Climate model data were downloaded from the CMIP3 and CMIP5 downscaled climate and hydrology projections archive at https://gdo-dcp.ucllnl.org/downscaled_cmip_projections/. See methods section of the manuscript for details on the specific parameters used to access the data from these research groups. (DOCX) [file pone.0219555.s002.docx]

**Supplemental Table S2. Research groups that contributed model ensembles to the downscaled GCM data used in this study.**

| **Group** | **Institute** | **Model** | **Number of Ensembles** |
| --- | --- | --- | --- |
| Commonwealth Scientific and Industrial Research Organization (CSIRO) and Bureau of Meteorology (BOM), Australia | CSIRO-BOM | ACCESS1.0 | 1 |
| Beijing Climate Center, China Meteorological Administration | BCC | BCC-CSM1.1 | 1 |
| Canadian Centre for Climate Modelling and Analysis | CCCMA | CanESM2 | 5 |
| National Center for Atmospheric Research | NCAR | CCSM4 | 2 |
| Community Earth System Model Contributors | NSF-DOE-NCAR | CESM1(BGC) | 1 |
| Centre National de Recherches Météorologiques / Centre Européen de Recherche et Formation Avancée en Calcul Scientifique | CNRM-CERFACS | CNRM-CM5 | 1 |
| Commonwealth Scientific and Industrial Research Organization in collaboration with Queensland Climate Change Centre of Excellence | CSIRO-QCCCE | CSIRO-Mk3.6.0 | 10 |
| NOAA Geophysical Fluid Dynamics Laboratory | NOAA GFDL | GFDL-CM3 | 1 |
|  |  | GFDL-ESM2G | 1 |
|  |  | GFDL-ESM2M | 1 |
| Institute for Numerical Mathematics | INM | INM-CM4 | 1 |
| Institut Pierre-Simon Laplace | IPSL | IPSL-CM5A-LR | 4 |
|  |  | IPSL-CM5A-MR | 1 |
| Japan Agency for Marine-Earth Science and Technology, Atmosphere and Ocean Research Institute (The University of Tokyo), and National Institute for Environmental Studies | MIROC | MIROC-ESM | 1 |
|  |  | MIROC-ESM-CHEM | 1 |
| Atmosphere and Ocean Research Institute (The University of Tokyo), National Institute for Environmental Studies, and Japan Agency for Marine-Earth Science and Technology | MIROC | MIROC5 | 3 |
| Max-Planck-Institut für Meteorologie (Max Planck Institute for Meteorology) | MPI-M | MPI-ESM-MR | 1 |
|  |  | MPI-ESM-LR | 3 |
| Meteorological Research Institute | MRI | MRI-CGCM3 | 1 |
| Norwegian Climate Centre | NCC | NorESM1-M | 1 |
