# Supplementary material for: Winter is (not) coming: Warming temperatures will affect the overwinter behavior and survival of blue crab
Source: PLoS One. 2019 Jul 25;14(7):e0219555. doi: 10.1371/journal.pone.0219555 (PMC6657847; doi:10.1371/journal.pone.0219555)
Supplement: S1 Appendix — (DOCX) [file pone.0219555.s003.docx]

**S1 Appendix.** R code for harmonic model, future temperature predictions, and future crab survival.

#set directories

indirectory="C:/Users/Hillary/Documents/Crab Model Data/"

infile="Crab Model R Data"

insheet="Sheet1"

#import data

pierdata=read_excel(paste(indirectory,infile,".xlsx",sep=""),sheet=insheet,col_names=TRUE, col_types=NULL)

#add some variants of date for later modelling

#calculate day of year from date

pierdata$DOY=as.numeric(pierdata$JulianDay)

pierdata$Year=as.numeric(pierdata$Year)

pierdata$tsYear=pierdata$Year-1937

pierdata$tsDay=julian(pierdata$Date, origin="1938-01-01",digits=2)

pierdata$tsDay=ceiling(pierdata$tsDay)

pierdata$tsDay=as.numeric(pierdata$tsDay)

########################################################################

#use entire dataset to get calibration and validation coefficients for harmonic model

#interpolate between all missing data points for water and air temp

library(zoo)

pierdata$CombinedWaterTemp=na.approx(pierdata$CorrectedWaterTemp, x = index(pierdata$CorrectedWaterTemp), maxgap=30, na.rm=FALSE)

#count number of observations per year after interpolation

pier.obs.water <- aggregate(cbind(count = CombinedWaterTemp) ~ Year,

data = pierdata,

FUN = function(x){NROW(x)})

pierdata$CombinedAirTemp=na.approx(pierdata$CombinedAirTemp, x = index(pierdata$CombinedAirTemp), maxgap=30, na.rm=FALSE)

pier.obs.air <- aggregate(cbind(count = CombinedAirTemp) ~ Year,

data = pierdata,

FUN = function(x){NROW(x)})

#remove 2 years with less than 350 observations for air and water temp (1977 and 1987)

pierdata$CombinedWaterTemp=na.approx(pierdata$CorrectedWaterTemp, x = index(pierdata$CorrectedWaterTemp), maxgap=30, na.rm=FALSE)

#count number of observations per year after interpolation

pierdata <- pierdata[ which(pierdata$Year!="1977" & pierdata$Year != "1987"),]

#create calibration and validation coefficients based on the first order harmonic model

library(lmodel2)

################################################

#add in some funtions

#create function that builds harmonic equation

harmonic<-function(m){

hold<-NULL

for (i in seq(1,m,1)){

hold[i]<-paste0('sin(2*pi*DOY/365*',i,')+cos(2*pi*DOY/365*',i, ')')

}

paste(hold, collapse='+')

}

Water.RMSE.Cal<-function(order,cal){

our.model<-paste('CombinedWaterTemp~tsDay +', harmonic(order))

final.model<-lm(as.formula(our.model),data=cal)

cal$preds<-predict(final.model,cal)

RMSE<-rmse(cal$preds, cal$CombinedWaterTemp, na.rm=TRUE)

}

Air.RMSE.Cal<-function(order,cal){

our.model<-paste('CombinedAirTemp~tsDay +', harmonic(order))

final.model<-lm(as.formula(our.model),data=cal)

cal$preds<-predict(final.model,cal)

RMSE<-rmse(cal$preds, cal$CombinedAirTemp, na.rm=TRUE)

}

Water.Coef<-function(order, cal){

our.model<-paste('CombinedWaterTemp~tsDay +', harmonic(order))

final.model<-lm(as.formula(our.model),data=cal)

coef(final.model)

}

Air.Coef<-function(order, cal){

our.model<-paste('CombinedAirTemp~tsDay +', harmonic(order))

final.model<-lm(as.formula(our.model),data=cal)

coef(final.model)

}

##########################

#predictive model

#first, get error of water predicting water and air predciting air in the whole pierdata set for the first order

order=1

Water.RMSE.All<-sapply(order,Water.RMSE.Cal, cal=pierdata)

Air.RMSE.All<-sapply(order,Air.RMSE.Cal, cal=pierdata)

#then, get coefficients for whole dataset for the first harmonic

Water.Cal.Coef1 <- Water.Coef(1,pierdata)

Water.Cal.Coef1 <- as.data.frame(Water.Cal.Coef1)

Air.Cal.Coef1 <- Air.Coef(1,pierdata)

Air.Cal.Coef1 <- as.data.frame(Air.Cal.Coef1)

Cal.Coef1 <- matrix(nrow=4,ncol=3)

Cal.Coef1[,1] <- Water.Cal.Coef1[,1]

Cal.Coef1[,2] <- Air.Cal.Coef1[,1]

Cal.Coef1 <- as.data.frame(Cal.Coef1)

Cal.Coef1[,3] <- Cal.Coef1[,1]/Cal.Coef1[,2]

colnames(Cal.Coef1) <- c("Water Coef","Air Coef","Ratio")

Ratio.Coef1 <- matrix(nrow=2,ncol=2)

Ratio.Coef1[,1] <- c(Cal.Coef1[3,3])

Ratio.Coef1[,2] <- c(Cal.Coef1[4,3])

Ratio.Coef1 <- as.data.frame(Ratio.Coef1)

colnames(Ratio.Coef1) <- c("sin","cos")

Air.Val.Coef1 <- Air.Coef(1,pierdata)

Air.Val.Coef1 <- as.data.frame(Air.Val.Coef1)

Air.Val.Coef.1 <- matrix(nrow=2,ncol=2)

Air.Val.Coef.1[,1] <- c(Air.Val.Coef1[3,1])

Air.Val.Coef.1[,2] <- c(Air.Val.Coef1[4,1])

Air.Val.Coef.1 <- as.data.frame(Air.Val.Coef.1)

colnames(Air.Val.Coef.1) <- c("sin","cos")

Water.Val.Coef1 <- Water.Coef(1,pierdata)

Water.Val.Coef1 <- as.data.frame(Water.Val.Coef1)

#use air temp and new ratio coef to predict water temp

harmonic.ratio.1<-function(m){

hold<-NULL

for (i in seq(1,m,1)){

hold[i]<-paste0('Air.Val.Coef.1[',i,',1]*Ratio.Coef1[',i,',1]*sin(2*pi*pierdata$DOY/365*',i,')+Air.Val.Coef.1[',i,',2]*Ratio.Coef1[',i,',2]*cos(2*pi*pierdata$DOY/365*',i, ')')

}

paste(hold, collapse='+')

}

harm<-harmonic.ratio.1(1)

Predict.Model.Ratio<-paste('Water.Val.Coef1[1,1]+Water.Val.Coef1[2,1]*pierdata$tsDay +', harm)

Predict.Model.Ratio

pierdata$WTPredictR.1 <- Water.Val.Coef1[1,1]+Water.Val.Coef1[2,1]*pierdata$tsDay + Air.Val.Coef.1[1,1]*Ratio.Coef1[1,1]*sin(2*pi*pierdata$DOY/365*1)+Air.Val.Coef.1[1,2]*Ratio.Coef1[1,2]*cos(2*pi*pierdata$DOY/365*1)

Water.RMSE.1 <- rmse(pierdata$WTPredictR.1, pierdata$CombinedWaterTemp, na.rm=TRUE)

#######################################################################################

###################################################################

#extend current trend in CBL pier data to get a conservative estimate of future temps

#make a new dataframe with tsDay and DOY from Jan 1, 2017 through Dec 31, 2099

library(tidyr)

library(date)

library(lubridate)

library(plyr)

#run water temp model to get slope and intercept

#number of obs is 24680 for water temp (for se to sd coversion)

our.model<-paste('CombinedWaterTemp~tsDay +', harmonic(1))

final.model<-lm(as.formula(our.model),data=pierdata)

summary(final.model)

Intercept.mean <- coef(summary(final.model))[1,1]

Intercept.sd <- coef(summary(final.model))[1,2]

Slope.mean <- coef(summary(final.model))[2,1]

Slope.sd <- coef(summary(final.model))[2,2]

#run air temp model to get sin and cos coef

#number of obs is 25026 for air temp (for se to sd conversion)

our.model<-paste('CombinedAirTemp~tsDay +', harmonic(1))

final.model<-lm(as.formula(our.model),data=pierdata)

summary(final.model)

sin.mean <- coef(summary(final.model))[3,1]

sin.sd <- coef(summary(final.model))[3,2]

cos.mean <- coef(summary(final.model))[4,1]

cos.sd <- coef(summary(final.model))[4,2]

#bootstrap through each term using mean and sd to get 50 combinations of coef for error for model

means <- rnorm(50,Intercept.mean,Intercept.sd)

bootstrap.coef <- as.data.frame(means)

colnames(bootstrap.coef) <- "Intercept"

bootstrap.coef$Slope <- rnorm(50,Slope.mean,Slope.sd)

bootstrap.coef$sin <- rnorm(50,sin.mean,sin.sd)

bootstrap.coef$cos <- rnorm(50,cos.mean,cos.sd)

#cycle through all coef to run the extension of the current trend 50 times

List=list()

for (i in seq(1,50,1)){

regress.model <- paste('bootstrap.coef[',i,',1]+bootstrap.coef[',i,',2]*pierdata.extend$tsDay +bootstrap.coef[',i,',3]*Ratio.Coef1[1,1]*sin(2*pi*pierdata.extend$DOY/365)+bootstrap.coef[',i,',4]*Ratio.Coef1[1,2]*cos(2*pi*pierdata.extend$DOY/365)')

List[[length(List)+1]] = regress.model

}

pierdata.extend <- seq(as.Date("2017/1/1"), as.Date("2099/12/31"), "days")

pierdata.extend <- as.data.frame(pierdata.extend)

colnames(pierdata.extend) <- "Date"

pierdata.extend$DOY <- yday(pierdata.extend$Date)

pierdata.extend$tsDay <- julian(pierdata.extend$Date, origin=as.Date("1938-01-01"))

pierdata.extend <- mutate(pierdata.extend, Date = ymd(Date), day = day(Date),

month = month(Date), year = year(Date))

pierdata.extend$WT1.1 <- bootstrap.coef[ 1 ,1]+bootstrap.coef[ 1 ,2]*pierdata.extend$tsDay +bootstrap.coef[ 1 ,3]*Ratio.Coef1[ 1 ,1]*sin(2*pi*pierdata.extend$DOY/365)+bootstrap.coef[ 1 ,4]*Ratio.Coef1[ 1 ,2]*cos(2*pi*pierdata.extend$DOY/365)

pierdata.extend$WT1.2 <- bootstrap.coef[ 2 ,1]+bootstrap.coef[ 2 ,2]*pierdata.extend$tsDay +bootstrap.coef[ 2 ,3]*Ratio.Coef1[ 1 ,1]*sin(2*pi*pierdata.extend$DOY/365)+bootstrap.coef[ 2 ,4]*Ratio.Coef1[ 1 ,2]*cos(2*pi*pierdata.extend$DOY/365)

pierdata.extend$WT1.3 <- bootstrap.coef[ 3 ,1]+bootstrap.coef[ 3 ,2]*pierdata.extend$tsDay +bootstrap.coef[ 3 ,3]*Ratio.Coef1[ 1 ,1]*sin(2*pi*pierdata.extend$DOY/365)+bootstrap.coef[ 3 ,4]*Ratio.Coef1[ 1 ,2]*cos(2*pi*pierdata.extend$DOY/365)

pierdata.extend$WT1.4 <- bootstrap.coef[ 4 ,1]+bootstrap.coef[ 4 ,2]*pierdata.extend$tsDay +bootstrap.coef[ 4 ,3]*Ratio.Coef1[ 1 ,1]*sin(2*pi*pierdata.extend$DOY/365)+bootstrap.coef[ 4 ,4]*Ratio.Coef1[ 1 ,2]*cos(2*pi*pierdata.extend$DOY/365)

pierdata.extend$WT1.5 <- bootstrap.coef[ 5 ,1]+bootstrap.coef[ 5 ,2]*pierdata.extend$tsDay +bootstrap.coef[ 5 ,3]*Ratio.Coef1[ 1 ,1]*sin(2*pi*pierdata.extend$DOY/365)+bootstrap.coef[ 5 ,4]*Ratio.Coef1[ 1 ,2]*cos(2*pi*pierdata.extend$DOY/365)

pierdata.extend$WT1.6 <- bootstrap.coef[ 6 ,1]+bootstrap.coef[ 6 ,2]*pierdata.extend$tsDay +bootstrap.coef[ 6 ,3]*Ratio.Coef1[ 1 ,1]*sin(2*pi*pierdata.extend$DOY/365)+bootstrap.coef[ 6 ,4]*Ratio.Coef1[ 1 ,2]*cos(2*pi*pierdata.extend$DOY/365)

pierdata.extend$WT1.7 <- bootstrap.coef[ 7 ,1]+bootstrap.coef[ 7 ,2]*pierdata.extend$tsDay +bootstrap.coef[ 7 ,3]*Ratio.Coef1[ 1 ,1]*sin(2*pi*pierdata.extend$DOY/365)+bootstrap.coef[ 7 ,4]*Ratio.Coef1[ 1 ,2]*cos(2*pi*pierdata.extend$DOY/365)

pierdata.extend$WT1.8 <- bootstrap.coef[ 8 ,1]+bootstrap.coef[ 8 ,2]*pierdata.extend$tsDay +bootstrap.coef[ 8 ,3]*Ratio.Coef1[ 1 ,1]*sin(2*pi*pierdata.extend$DOY/365)+bootstrap.coef[ 8 ,4]*Ratio.Coef1[ 1 ,2]*cos(2*pi*pierdata.extend$DOY/365)

pierdata.extend$WT1.9 <- bootstrap.coef[ 9 ,1]+bootstrap.coef[ 9 ,2]*pierdata.extend$tsDay +bootstrap.coef[ 9 ,3]*Ratio.Coef1[1,1]*sin(2*pi*pierdata.extend$DOY/365)+bootstrap.coef[ 9 ,4]*Ratio.Coef1[ 1 ,2]*cos(2*pi*pierdata.extend$DOY/365)

pierdata.extend$WT1.10 <- bootstrap.coef[ 10 ,1]+bootstrap.coef[ 10 ,2]*pierdata.extend$tsDay +bootstrap.coef[ 10 ,3]*Ratio.Coef1[ 1 ,1]*sin(2*pi*pierdata.extend$DOY/365)+bootstrap.coef[ 10 ,4]*Ratio.Coef1[ 1 ,2]*cos(2*pi*pierdata.extend$DOY/365)

pierdata.extend$WT1.11 <- bootstrap.coef[ 11 ,1]+bootstrap.coef[ 11 ,2]*pierdata.extend$tsDay +bootstrap.coef[ 11 ,3]*Ratio.Coef1[ 1 ,1]*sin(2*pi*pierdata.extend$DOY/365)+bootstrap.coef[ 11 ,4]*Ratio.Coef1[ 1 ,2]*cos(2*pi*pierdata.extend$DOY/365)

pierdata.extend$WT1.12 <- bootstrap.coef[ 12 ,1]+bootstrap.coef[ 12 ,2]*pierdata.extend$tsDay +bootstrap.coef[ 12 ,3]*Ratio.Coef1[ 1 ,1]*sin(2*pi*pierdata.extend$DOY/365)+bootstrap.coef[ 12 ,4]*Ratio.Coef1[ 1 ,2]*cos(2*pi*pierdata.extend$DOY/365)

pierdata.extend$WT1.13 <- bootstrap.coef[ 13 ,1]+bootstrap.coef[ 13 ,2]*pierdata.extend$tsDay +bootstrap.coef[ 13 ,3]*Ratio.Coef1[ 1 ,1]*sin(2*pi*pierdata.extend$DOY/365)+bootstrap.coef[ 13 ,4]*Ratio.Coef1[ 1 ,2]*cos(2*pi*pierdata.extend$DOY/365)

pierdata.extend$WT1.14 <- bootstrap.coef[ 14 ,1]+bootstrap.coef[ 14 ,2]*pierdata.extend$tsDay +bootstrap.coef[ 14 ,3]*Ratio.Coef1[ 1 ,1]*sin(2*pi*pierdata.extend$DOY/365)+bootstrap.coef[ 14 ,4]*Ratio.Coef1[ 1 ,2]*cos(2*pi*pierdata.extend$DOY/365)

pierdata.extend$WT1.15 <- bootstrap.coef[ 15 ,1]+bootstrap.coef[ 15 ,2]*pierdata.extend$tsDay +bootstrap.coef[ 15 ,3]*Ratio.Coef1[ 1 ,1]*sin(2*pi*pierdata.extend$DOY/365)+bootstrap.coef[ 15 ,4]*Ratio.Coef1[ 1 ,2]*cos(2*pi*pierdata.extend$DOY/365)

pierdata.extend$WT1.16 <- bootstrap.coef[ 16 ,1]+bootstrap.coef[ 16 ,2]*pierdata.extend$tsDay +bootstrap.coef[ 16 ,3]*Ratio.Coef1[ 1 ,1]*sin(2*pi*pierdata.extend$DOY/365)+bootstrap.coef[ 16 ,4]*Ratio.Coef1[ 1 ,2]*cos(2*pi*pierdata.extend$DOY/365)

pierdata.extend$WT1.17 <- bootstrap.coef[ 17 ,1]+bootstrap.coef[ 17 ,2]*pierdata.extend$tsDay +bootstrap.coef[ 17 ,3]*Ratio.Coef1[ 1 ,1]*sin(2*pi*pierdata.extend$DOY/365)+bootstrap.coef[ 17 ,4]*Ratio.Coef1[ 1 ,2]*cos(2*pi*pierdata.extend$DOY/365)

pierdata.extend$WT1.18 <- bootstrap.coef[ 18 ,1]+bootstrap.coef[ 18 ,2]*pierdata.extend$tsDay +bootstrap.coef[ 18 ,3]*Ratio.Coef1[ 1 ,1]*sin(2*pi*pierdata.extend$DOY/365)+bootstrap.coef[ 18 ,4]*Ratio.Coef1[ 1 ,2]*cos(2*pi*pierdata.extend$DOY/365)

pierdata.extend$WT1.19 <- bootstrap.coef[ 19 ,1]+bootstrap.coef[ 19 ,2]*pierdata.extend$tsDay +bootstrap.coef[ 19 ,3]*Ratio.Coef1[ 1 ,1]*sin(2*pi*pierdata.extend$DOY/365)+bootstrap.coef[ 19 ,4]*Ratio.Coef1[ 1 ,2]*cos(2*pi*pierdata.extend$DOY/365)

pierdata.extend$WT1.20 <- bootstrap.coef[ 20 ,1]+bootstrap.coef[ 20 ,2]*pierdata.extend$tsDay +bootstrap.coef[ 20 ,3]*Ratio.Coef1[ 1 ,1]*sin(2*pi*pierdata.extend$DOY/365)+bootstrap.coef[ 20 ,4]*Ratio.Coef1[ 1 ,2]*cos(2*pi*pierdata.extend$DOY/365)

pierdata.extend$WT1.21 <- bootstrap.coef[ 21 ,1]+bootstrap.coef[ 21 ,2]*pierdata.extend$tsDay +bootstrap.coef[ 21 ,3]*Ratio.Coef1[ 1 ,1]*sin(2*pi*pierdata.extend$DOY/365)+bootstrap.coef[ 21 ,4]*Ratio.Coef1[ 1 ,2]*cos(2*pi*pierdata.extend$DOY/365)

pierdata.extend$WT1.22 <- bootstrap.coef[ 22 ,1]+bootstrap.coef[ 22 ,2]*pierdata.extend$tsDay +bootstrap.coef[ 22 ,3]*Ratio.Coef1[ 1 ,1]*sin(2*pi*pierdata.extend$DOY/365)+bootstrap.coef[ 22 ,4]*Ratio.Coef1[ 1 ,2]*cos(2*pi*pierdata.extend$DOY/365)

pierdata.extend$WT1.23 <- bootstrap.coef[ 23 ,1]+bootstrap.coef[ 23 ,2]*pierdata.extend$tsDay +bootstrap.coef[ 23 ,3]*Ratio.Coef1[ 1 ,1]*sin(2*pi*pierdata.extend$DOY/365)+bootstrap.coef[ 23 ,4]*Ratio.Coef1[ 1 ,2]*cos(2*pi*pierdata.extend$DOY/365)

pierdata.extend$WT1.24 <- bootstrap.coef[ 24 ,1]+bootstrap.coef[ 24 ,2]*pierdata.extend$tsDay +bootstrap.coef[ 24 ,3]*Ratio.Coef1[ 1 ,1]*sin(2*pi*pierdata.extend$DOY/365)+bootstrap.coef[ 24 ,4]*Ratio.Coef1[ 1 ,2]*cos(2*pi*pierdata.extend$DOY/365)

pierdata.extend$WT1.25 <- bootstrap.coef[ 25 ,1]+bootstrap.coef[ 25 ,2]*pierdata.extend$tsDay +bootstrap.coef[ 25 ,3]*Ratio.Coef1[ 1 ,1]*sin(2*pi*pierdata.extend$DOY/365)+bootstrap.coef[ 25 ,4]*Ratio.Coef1[ 1 ,2]*cos(2*pi*pierdata.extend$DOY/365)

pierdata.extend$WT1.26 <- bootstrap.coef[ 26 ,1]+bootstrap.coef[ 26 ,2]*pierdata.extend$tsDay +bootstrap.coef[ 26 ,3]*Ratio.Coef1[ 1 ,1]*sin(2*pi*pierdata.extend$DOY/365)+bootstrap.coef[ 26 ,4]*Ratio.Coef1[ 1 ,2]*cos(2*pi*pierdata.extend$DOY/365)

pierdata.extend$WT1.27 <- bootstrap.coef[ 27 ,1]+bootstrap.coef[ 27 ,2]*pierdata.extend$tsDay +bootstrap.coef[ 27 ,3]*Ratio.Coef1[ 1 ,1]*sin(2*pi*pierdata.extend$DOY/365)+bootstrap.coef[ 27 ,4]*Ratio.Coef1[ 1 ,2]*cos(2*pi*pierdata.extend$DOY/365)

pierdata.extend$WT1.28 <- bootstrap.coef[ 28 ,1]+bootstrap.coef[ 28 ,2]*pierdata.extend$tsDay +bootstrap.coef[ 28 ,3]*Ratio.Coef1[ 1 ,1]*sin(2*pi*pierdata.extend$DOY/365)+bootstrap.coef[ 28 ,4]*Ratio.Coef1[ 1 ,2]*cos(2*pi*pierdata.extend$DOY/365)

pierdata.extend$WT1.29 <- bootstrap.coef[ 29 ,1]+bootstrap.coef[ 29 ,2]*pierdata.extend$tsDay +bootstrap.coef[ 29 ,3]*Ratio.Coef1[ 1 ,1]*sin(2*pi*pierdata.extend$DOY/365)+bootstrap.coef[ 29 ,4]*Ratio.Coef1[ 1 ,2]*cos(2*pi*pierdata.extend$DOY/365)

pierdata.extend$WT1.30 <- bootstrap.coef[ 30 ,1]+bootstrap.coef[ 30 ,2]*pierdata.extend$tsDay +bootstrap.coef[ 30 ,3]*Ratio.Coef1[ 1 ,1]*sin(2*pi*pierdata.extend$DOY/365)+bootstrap.coef[ 30 ,4]*Ratio.Coef1[ 1 ,2]*cos(2*pi*pierdata.extend$DOY/365)

pierdata.extend$WT1.31 <- bootstrap.coef[ 31 ,1]+bootstrap.coef[ 31 ,2]*pierdata.extend$tsDay +bootstrap.coef[ 31 ,3]*Ratio.Coef1[ 1 ,1]*sin(2*pi*pierdata.extend$DOY/365)+bootstrap.coef[ 31 ,4]*Ratio.Coef1[ 1 ,2]*cos(2*pi*pierdata.extend$DOY/365)

pierdata.extend$WT1.32 <- bootstrap.coef[ 32 ,1]+bootstrap.coef[ 32 ,2]*pierdata.extend$tsDay +bootstrap.coef[ 32 ,3]*Ratio.Coef1[ 1 ,1]*sin(2*pi*pierdata.extend$DOY/365)+bootstrap.coef[ 32 ,4]*Ratio.Coef1[ 1 ,2]*cos(2*pi*pierdata.extend$DOY/365)

pierdata.extend$WT1.33 <- bootstrap.coef[ 33 ,1]+bootstrap.coef[ 33 ,2]*pierdata.extend$tsDay +bootstrap.coef[ 33 ,3]*Ratio.Coef1[ 1 ,1]*sin(2*pi*pierdata.extend$DOY/365)+bootstrap.coef[ 33 ,4]*Ratio.Coef1[ 1 ,2]*cos(2*pi*pierdata.extend$DOY/365)

pierdata.extend$WT1.34 <- bootstrap.coef[ 34 ,1]+bootstrap.coef[ 34 ,2]*pierdata.extend$tsDay +bootstrap.coef[ 34 ,3]*Ratio.Coef1[ 1 ,1]*sin(2*pi*pierdata.extend$DOY/365)+bootstrap.coef[ 34 ,4]*Ratio.Coef1[ 1 ,2]*cos(2*pi*pierdata.extend$DOY/365)

pierdata.extend$WT1.35 <- bootstrap.coef[ 35 ,1]+bootstrap.coef[ 35 ,2]*pierdata.extend$tsDay +bootstrap.coef[ 35 ,3]*Ratio.Coef1[ 1 ,1]*sin(2*pi*pierdata.extend$DOY/365)+bootstrap.coef[ 35 ,4]*Ratio.Coef1[ 1 ,2]*cos(2*pi*pierdata.extend$DOY/365)

pierdata.extend$WT1.36 <- bootstrap.coef[ 36 ,1]+bootstrap.coef[ 36 ,2]*pierdata.extend$tsDay +bootstrap.coef[ 36 ,3]*Ratio.Coef1[ 1 ,1]*sin(2*pi*pierdata.extend$DOY/365)+bootstrap.coef[ 36 ,4]*Ratio.Coef1[ 1 ,2]*cos(2*pi*pierdata.extend$DOY/365)

pierdata.extend$WT1.37 <- bootstrap.coef[ 37 ,1]+bootstrap.coef[ 37 ,2]*pierdata.extend$tsDay +bootstrap.coef[ 37 ,3]*Ratio.Coef1[ 1 ,1]*sin(2*pi*pierdata.extend$DOY/365)+bootstrap.coef[ 37 ,4]*Ratio.Coef1[ 1 ,2]*cos(2*pi*pierdata.extend$DOY/365)

pierdata.extend$WT1.38 <- bootstrap.coef[ 38 ,1]+bootstrap.coef[ 38 ,2]*pierdata.extend$tsDay +bootstrap.coef[ 38 ,3]*Ratio.Coef1[ 1 ,1]*sin(2*pi*pierdata.extend$DOY/365)+bootstrap.coef[ 38 ,4]*Ratio.Coef1[1 ,2]*cos(2*pi*pierdata.extend$DOY/365)

pierdata.extend$WT1.39 <- bootstrap.coef[ 39 ,1]+bootstrap.coef[ 39 ,2]*pierdata.extend$tsDay +bootstrap.coef[ 39 ,3]*Ratio.Coef1[ 1 ,1]*sin(2*pi*pierdata.extend$DOY/365)+bootstrap.coef[ 39 ,4]*Ratio.Coef1[ 1 ,2]*cos(2*pi*pierdata.extend$DOY/365)

pierdata.extend$WT1.40 <- bootstrap.coef[ 40 ,1]+bootstrap.coef[ 40 ,2]*pierdata.extend$tsDay +bootstrap.coef[ 40 ,3]*Ratio.Coef1[ 1 ,1]*sin(2*pi*pierdata.extend$DOY/365)+bootstrap.coef[ 40 ,4]*Ratio.Coef1[ 1 ,2]*cos(2*pi*pierdata.extend$DOY/365)

pierdata.extend$WT1.41 <- bootstrap.coef[ 41 ,1]+bootstrap.coef[ 41 ,2]*pierdata.extend$tsDay +bootstrap.coef[ 41 ,3]*Ratio.Coef1[ 1 ,1]*sin(2*pi*pierdata.extend$DOY/365)+bootstrap.coef[ 41 ,4]*Ratio.Coef1[1 ,2]*cos(2*pi*pierdata.extend$DOY/365)

pierdata.extend$WT1.42 <- bootstrap.coef[ 42 ,1]+bootstrap.coef[ 42 ,2]*pierdata.extend$tsDay +bootstrap.coef[ 42 ,3]*Ratio.Coef1[ 1 ,1]*sin(2*pi*pierdata.extend$DOY/365)+bootstrap.coef[ 42 ,4]*Ratio.Coef1[ 1 ,2]*cos(2*pi*pierdata.extend$DOY/365)

pierdata.extend$WT1.43 <- bootstrap.coef[ 43 ,1]+bootstrap.coef[ 43 ,2]*pierdata.extend$tsDay +bootstrap.coef[ 43 ,3]*Ratio.Coef1[ 1 ,1]*sin(2*pi*pierdata.extend$DOY/365)+bootstrap.coef[ 43 ,4]*Ratio.Coef1[ 1 ,2]*cos(2*pi*pierdata.extend$DOY/365)

pierdata.extend$WT1.44 <- bootstrap.coef[ 44 ,1]+bootstrap.coef[ 44 ,2]*pierdata.extend$tsDay +bootstrap.coef[ 44 ,3]*Ratio.Coef1[ 1 ,1]*sin(2*pi*pierdata.extend$DOY/365)+bootstrap.coef[ 44 ,4]*Ratio.Coef1[ 1 ,2]*cos(2*pi*pierdata.extend$DOY/365)

pierdata.extend$WT1.45 <- bootstrap.coef[ 45 ,1]+bootstrap.coef[ 45 ,2]*pierdata.extend$tsDay +bootstrap.coef[ 45 ,3]*Ratio.Coef1[ 1 ,1]*sin(2*pi*pierdata.extend$DOY/365)+bootstrap.coef[ 45 ,4]*Ratio.Coef1[ 1 ,2]*cos(2*pi*pierdata.extend$DOY/365)

pierdata.extend$WT1.46 <- bootstrap.coef[ 46 ,1]+bootstrap.coef[ 46 ,2]*pierdata.extend$tsDay +bootstrap.coef[ 46 ,3]*Ratio.Coef1[ 1 ,1]*sin(2*pi*pierdata.extend$DOY/365)+bootstrap.coef[ 46 ,4]*Ratio.Coef1[ 1 ,2]*cos(2*pi*pierdata.extend$DOY/365)

pierdata.extend$WT1.47 <- bootstrap.coef[ 47 ,1]+bootstrap.coef[ 47 ,2]*pierdata.extend$tsDay +bootstrap.coef[ 47 ,3]*Ratio.Coef1[ 1 ,1]*sin(2*pi*pierdata.extend$DOY/365)+bootstrap.coef[ 47 ,4]*Ratio.Coef1[ 1 ,2]*cos(2*pi*pierdata.extend$DOY/365)

pierdata.extend$WT1.48 <- bootstrap.coef[ 48 ,1]+bootstrap.coef[ 48 ,2]*pierdata.extend$tsDay +bootstrap.coef[ 48 ,3]*Ratio.Coef1[ 1 ,1]*sin(2*pi*pierdata.extend$DOY/365)+bootstrap.coef[ 48 ,4]*Ratio.Coef1[ 1 ,2]*cos(2*pi*pierdata.extend$DOY/365)

pierdata.extend$WT1.49 <- bootstrap.coef[ 49 ,1]+bootstrap.coef[ 49 ,2]*pierdata.extend$tsDay +bootstrap.coef[ 49 ,3]*Ratio.Coef1[ 1 ,1]*sin(2*pi*pierdata.extend$DOY/365)+bootstrap.coef[ 49 ,4]*Ratio.Coef1[ 1 ,2]*cos(2*pi*pierdata.extend$DOY/365)

pierdata.extend$WT1.50 <- bootstrap.coef[ 50 ,1]+bootstrap.coef[ 50 ,2]*pierdata.extend$tsDay +bootstrap.coef[ 50 ,3]*Ratio.Coef1[ 1 ,1]*sin(2*pi*pierdata.extend$DOY/365)+bootstrap.coef[ 50 ,4]*Ratio.Coef1[ 1 ,2]*cos(2*pi*pierdata.extend$DOY/365)

pierdata.extend$mean <- rowMeans(pierdata.extend[,7:56])

pierdata.extend2 <- pierdata.extend[,7:56]

pierdata.extend2$Year <- pierdata.extend$year

winter.by.year.extend <- sapply(split(pierdata.extend2, pierdata.extend2$Year),

function(x) apply(x,2, function(x) length(x[x<9])))

winter.by.year.extend <- as.data.frame(winter.by.year.extend)

winter.by.year.extend <- t(winter.by.year.extend)

winter.by.year.extend <- as.data.frame(winter.by.year.extend)

winter.by.year.extend$Year <- seq(2017,2099,1)

#winter.by.year.extend <- subset(winter.by.year.extend,Year<2100&Year>2017)

winter.by.year.extend$mean <- rowMeans(winter.by.year.extend[,1:50])

library(matrixStats)

wt.matrix2.ex <- data.matrix(winter.by.year.extend[,1:50],rownames.force = NA)

winter.by.year.extend$SD <- rowSds(wt.matrix2.ex)

winter.by.year.extend$Min <- rowMins(wt.matrix2.ex)

winter.by.year.extend$Max <- rowMaxs(wt.matrix2.ex)

winter.by.year.extend$SD1 <- winter.by.year.extend$mean+winter.by.year.extend$SD

winter.by.year.extend$SD2 <- winter.by.year.extend$mean-winter.by.year.extend$SD

#######################################

#use reference interval from Maloney paper (1961-1990) to calculate average year for the past

#compare that to an average year in the future reference period (2070-2099)

pierdata.subset <- subset(pierdata,Year<1991 & Year>1960)

mean.temps.water <- tapply(pierdata.subset$CombinedWaterTemp, pierdata.subset$DOY, mean, na.rm=TRUE)

sd.temps.water <- tapply(pierdata.subset$CombinedWaterTemp, pierdata.subset$DOY, sd, na.rm=TRUE)

mean.temps <- as.data.frame(mean.temps.water)

mean.temps[,2] <- sd.temps.water

mean.temps[,3] <- seq(1,366,1)

colnames(mean.temps) <- c("RefWaterMean","RefWaterSD","DOY")

mean.temps$RefWaterSDLine <- mean.temps$RefWaterMean+mean.temps$RefWaterSD

mean.temps$RefWaterSDLine2 <- mean.temps$RefWaterMean-mean.temps$RefWaterSD

#create subset of predicted pier data with just trend extended and then do the same calcs as above

pierdata.extend3 <- subset(pierdata.extend, year>2069)

mean.temps.water.pred1 <- tapply(pierdata.extend3$mean, pierdata.extend3$DOY, mean, na.rm=TRUE)

sd.temps.water.pred1 <- tapply(pierdata.extend3$mean, pierdata.extend3$DOY, sd, na.rm=TRUE)

mean.temps.pred <- as.data.frame(mean.temps.water.pred1)

colnames(mean.temps.pred) <- "WT1"

mean.temps.pred$SD1.W <- sd.temps.water.pred1

mean.temps.pred$DOY <- seq(1,366,1)

mean.temps.pred$WaterSDLine1.1 <- mean.temps.pred$WT1+mean.temps.pred$SD1.W

mean.temps.pred$WaterSDLine1.2 <- mean.temps.pred$WT1-mean.temps.pred$SD1.W

##############################################

#use CIMP5 data to calculate trend in air temp increase and apply our model to determine water temp

#do for each input CMIP model separately

CIMP5.daily <- read.csv("CIMP5.daily.all.data.csv",head=T)

CIMP5.daily <- subset(CIMP5.daily,CIMP5.daily$JulianDate.1<59169)

#create a data frame with only the air temp data to run regressions for coefficients

CIMP5.daily2 <- CIMP5.daily[,8:48]

model=NULL

for (i in seq(1,41,1)){

model[i]<-paste0('V',i,'~JulianDate')

}

final.model=NULL

for (i in seq(1,41,1)){

final.model[i]<-lm(as.formula(model[i]),data=CIMP5.daily)

}

final.model2 <- as.data.frame(final.model)

#use air temp and ratio coefficients from pier data to predict water temp in future

#use slope and intercept of linear regression of daily CIMP air temp data

harmonic.ratio.all.CIMP1D<-function(m){

hold<-NULL

for (i in seq(1,m,1)){

hold[i]<-paste0('Air.Val.Coef.1[',i,',1]*Ratio.Coef1[',i,',1]*sin(2*pi*CIMP5.daily$DOY/365*',i,')+Air.Val.Coef.1[',i,',2]*Ratio.Coef1[',i,',2]*cos(2*pi*CIMP5.daily$DOY/365*',i, ')')

}

paste(hold, collapse='+')

}

harm1.D<-harmonic.ratio.all.CIMP1D(1)

List=list()

for (i in seq(1,41,1)){

regress.model <- paste('final.model2[1,',i,']+final.model2[2,',i,']*CIMP5.daily$JulianDate.1 +', harm1.D)

List[[length(List)+1]] = regress.model

}

CIMP5.daily$WT1 <- final.model2[1, 1 ]+final.model2[2, 1 ]*CIMP5.daily$JulianDate.1 + Air.Val.Coef.1[1,1]*Ratio.Coef1[1,1]*sin(2*pi*CIMP5.daily$DOY/365*1)+Air.Val.Coef.1[1,2]*Ratio.Coef1[1,2]*cos(2*pi*CIMP5.daily$DOY/365*1)

CIMP5.daily$WT2 <- final.model2[1, 2 ]+final.model2[2, 2 ]*CIMP5.daily$JulianDate.1 + Air.Val.Coef.1[1,1]*Ratio.Coef1[1,1]*sin(2*pi*CIMP5.daily$DOY/365*1)+Air.Val.Coef.1[1,2]*Ratio.Coef1[1,2]*cos(2*pi*CIMP5.daily$DOY/365*1)

CIMP5.daily$WT3 <- final.model2[1, 3 ]+final.model2[2, 3 ]*CIMP5.daily$JulianDate.1 + Air.Val.Coef.1[1,1]*Ratio.Coef1[1,1]*sin(2*pi*CIMP5.daily$DOY/365*1)+Air.Val.Coef.1[1,2]*Ratio.Coef1[1,2]*cos(2*pi*CIMP5.daily$DOY/365*1)

CIMP5.daily$WT4 <- final.model2[1, 4 ]+final.model2[2, 4 ]*CIMP5.daily$JulianDate.1 + Air.Val.Coef.1[1,1]*Ratio.Coef1[1,1]*sin(2*pi*CIMP5.daily$DOY/365*1)+Air.Val.Coef.1[1,2]*Ratio.Coef1[1,2]*cos(2*pi*CIMP5.daily$DOY/365*1)

CIMP5.daily$WT5 <- final.model2[1, 5 ]+final.model2[2, 5 ]*CIMP5.daily$JulianDate.1 + Air.Val.Coef.1[1,1]*Ratio.Coef1[1,1]*sin(2*pi*CIMP5.daily$DOY/365*1)+Air.Val.Coef.1[1,2]*Ratio.Coef1[1,2]*cos(2*pi*CIMP5.daily$DOY/365*1)

CIMP5.daily$WT6 <- final.model2[1, 6 ]+final.model2[2, 6 ]*CIMP5.daily$JulianDate.1 + Air.Val.Coef.1[1,1]*Ratio.Coef1[1,1]*sin(2*pi*CIMP5.daily$DOY/365*1)+Air.Val.Coef.1[1,2]*Ratio.Coef1[1,2]*cos(2*pi*CIMP5.daily$DOY/365*1)

CIMP5.daily$WT7 <- final.model2[1, 7 ]+final.model2[2, 7 ]*CIMP5.daily$JulianDate.1 + Air.Val.Coef.1[1,1]*Ratio.Coef1[1,1]*sin(2*pi*CIMP5.daily$DOY/365*1)+Air.Val.Coef.1[1,2]*Ratio.Coef1[1,2]*cos(2*pi*CIMP5.daily$DOY/365*1)

CIMP5.daily$WT8 <- final.model2[1, 8 ]+final.model2[2, 8 ]*CIMP5.daily$JulianDate.1 + Air.Val.Coef.1[1,1]*Ratio.Coef1[1,1]*sin(2*pi*CIMP5.daily$DOY/365*1)+Air.Val.Coef.1[1,2]*Ratio.Coef1[1,2]*cos(2*pi*CIMP5.daily$DOY/365*1)

CIMP5.daily$WT9 <- final.model2[1, 9 ]+final.model2[2, 9 ]*CIMP5.daily$JulianDate.1 + Air.Val.Coef.1[1,1]*Ratio.Coef1[1,1]*sin(2*pi*CIMP5.daily$DOY/365*1)+Air.Val.Coef.1[1,2]*Ratio.Coef1[1,2]*cos(2*pi*CIMP5.daily$DOY/365*1)

CIMP5.daily$WT10 <-final.model2[1, 10 ]+final.model2[2, 10 ]*CIMP5.daily$JulianDate.1 + Air.Val.Coef.1[1,1]*Ratio.Coef1[1,1]*sin(2*pi*CIMP5.daily$DOY/365*1)+Air.Val.Coef.1[1,2]*Ratio.Coef1[1,2]*cos(2*pi*CIMP5.daily$DOY/365*1)

CIMP5.daily$WT11 <- final.model2[1, 11 ]+final.model2[2, 11 ]*CIMP5.daily$JulianDate.1 + Air.Val.Coef.1[1,1]*Ratio.Coef1[1,1]*sin(2*pi*CIMP5.daily$DOY/365*1)+Air.Val.Coef.1[1,2]*Ratio.Coef1[1,2]*cos(2*pi*CIMP5.daily$DOY/365*1)

CIMP5.daily$WT12 <- final.model2[1, 12 ]+final.model2[2, 12 ]*CIMP5.daily$JulianDate.1 + Air.Val.Coef.1[1,1]*Ratio.Coef1[1,1]*sin(2*pi*CIMP5.daily$DOY/365*1)+Air.Val.Coef.1[1,2]*Ratio.Coef1[1,2]*cos(2*pi*CIMP5.daily$DOY/365*1)

CIMP5.daily$WT13 <- final.model2[1, 13]+final.model2[2, 13 ]*CIMP5.daily$JulianDate.1 + Air.Val.Coef.1[1,1]*Ratio.Coef1[1,1]*sin(2*pi*CIMP5.daily$DOY/365*1)+Air.Val.Coef.1[1,2]*Ratio.Coef1[1,2]*cos(2*pi*CIMP5.daily$DOY/365*1)

CIMP5.daily$WT14 <- final.model2[1, 14]+final.model2[2, 14 ]*CIMP5.daily$JulianDate.1 + Air.Val.Coef.1[1,1]*Ratio.Coef1[1,1]*sin(2*pi*CIMP5.daily$DOY/365*1)+Air.Val.Coef.1[1,2]*Ratio.Coef1[1,2]*cos(2*pi*CIMP5.daily$DOY/365*1)

CIMP5.daily$WT15 <- final.model2[1, 15]+final.model2[2, 15 ]*CIMP5.daily$JulianDate.1 + Air.Val.Coef.1[1,1]*Ratio.Coef1[1,1]*sin(2*pi*CIMP5.daily$DOY/365*1)+Air.Val.Coef.1[1,2]*Ratio.Coef1[1,2]*cos(2*pi*CIMP5.daily$DOY/365*1)

CIMP5.daily$WT16 <- final.model2[1, 16]+final.model2[2, 16]*CIMP5.daily$JulianDate.1 + Air.Val.Coef.1[1,1]*Ratio.Coef1[1,1]*sin(2*pi*CIMP5.daily$DOY/365*1)+Air.Val.Coef.1[1,2]*Ratio.Coef1[1,2]*cos(2*pi*CIMP5.daily$DOY/365*1)

CIMP5.daily$WT17 <- final.model2[1, 17]+final.model2[2, 17]*CIMP5.daily$JulianDate.1 + Air.Val.Coef.1[1,1]*Ratio.Coef1[1,1]*sin(2*pi*CIMP5.daily$DOY/365*1)+Air.Val.Coef.1[1,2]*Ratio.Coef1[1,2]*cos(2*pi*CIMP5.daily$DOY/365*1)

CIMP5.daily$WT18 <- final.model2[1, 18]+final.model2[2, 18]*CIMP5.daily$JulianDate.1 + Air.Val.Coef.1[1,1]*Ratio.Coef1[1,1]*sin(2*pi*CIMP5.daily$DOY/365*1)+Air.Val.Coef.1[1,2]*Ratio.Coef1[1,2]*cos(2*pi*CIMP5.daily$DOY/365*1)

CIMP5.daily$WT19 <- final.model2[1, 19]+final.model2[2, 19]*CIMP5.daily$JulianDate.1 + Air.Val.Coef.1[1,1]*Ratio.Coef1[1,1]*sin(2*pi*CIMP5.daily$DOY/365*1)+Air.Val.Coef.1[1,2]*Ratio.Coef1[1,2]*cos(2*pi*CIMP5.daily$DOY/365*1)

CIMP5.daily$WT20 <- final.model2[1, 20 ]+final.model2[2, 20 ]*CIMP5.daily$JulianDate.1 + Air.Val.Coef.1[1,1]*Ratio.Coef1[1,1]*sin(2*pi*CIMP5.daily$DOY/365*1)+Air.Val.Coef.1[1,2]*Ratio.Coef1[1,2]*cos(2*pi*CIMP5.daily$DOY/365*1)

CIMP5.daily$WT21 <- final.model2[1, 21 ]+final.model2[2, 21 ]*CIMP5.daily$JulianDate.1 + Air.Val.Coef.1[1,1]*Ratio.Coef1[1,1]*sin(2*pi*CIMP5.daily$DOY/365*1)+Air.Val.Coef.1[1,2]*Ratio.Coef1[1,2]*cos(2*pi*CIMP5.daily$DOY/365*1)

CIMP5.daily$WT22 <- final.model2[1, 22]+final.model2[2, 22 ]*CIMP5.daily$JulianDate.1 + Air.Val.Coef.1[1,1]*Ratio.Coef1[1,1]*sin(2*pi*CIMP5.daily$DOY/365*1)+Air.Val.Coef.1[1,2]*Ratio.Coef1[1,2]*cos(2*pi*CIMP5.daily$DOY/365*1)

CIMP5.daily$WT23 <- final.model2[1, 23 ]+final.model2[2, 23 ]*CIMP5.daily$JulianDate.1 + Air.Val.Coef.1[1,1]*Ratio.Coef1[1,1]*sin(2*pi*CIMP5.daily$DOY/365*1)+Air.Val.Coef.1[1,2]*Ratio.Coef1[1,2]*cos(2*pi*CIMP5.daily$DOY/365*1)

CIMP5.daily$WT24 <- final.model2[1, 24 ]+final.model2[2, 24 ]*CIMP5.daily$JulianDate.1 + Air.Val.Coef.1[1,1]*Ratio.Coef1[1,1]*sin(2*pi*CIMP5.daily$DOY/365*1)+Air.Val.Coef.1[1,2]*Ratio.Coef1[1,2]*cos(2*pi*CIMP5.daily$DOY/365*1)

CIMP5.daily$WT25 <- final.model2[1, 25 ]+final.model2[2, 25 ]*CIMP5.daily$JulianDate.1 + Air.Val.Coef.1[1,1]*Ratio.Coef1[1,1]*sin(2*pi*CIMP5.daily$DOY/365*1)+Air.Val.Coef.1[1,2]*Ratio.Coef1[1,2]*cos(2*pi*CIMP5.daily$DOY/365*1)

CIMP5.daily$WT26 <- final.model2[1, 26 ]+final.model2[2, 26 ]*CIMP5.daily$JulianDate.1 + Air.Val.Coef.1[1,1]*Ratio.Coef1[1,1]*sin(2*pi*CIMP5.daily$DOY/365*1)+Air.Val.Coef.1[1,2]*Ratio.Coef1[1,2]*cos(2*pi*CIMP5.daily$DOY/365*1)

CIMP5.daily$WT27 <- final.model2[1, 27 ]+final.model2[2, 27 ]*CIMP5.daily$JulianDate.1 + Air.Val.Coef.1[1,1]*Ratio.Coef1[1,1]*sin(2*pi*CIMP5.daily$DOY/365*1)+Air.Val.Coef.1[1,2]*Ratio.Coef1[1,2]*cos(2*pi*CIMP5.daily$DOY/365*1)

CIMP5.daily$WT28 <- final.model2[1, 28 ]+final.model2[2, 28 ]*CIMP5.daily$JulianDate.1 + Air.Val.Coef.1[1,1]*Ratio.Coef1[1,1]*sin(2*pi*CIMP5.daily$DOY/365*1)+Air.Val.Coef.1[1,2]*Ratio.Coef1[1,2]*cos(2*pi*CIMP5.daily$DOY/365*1)

CIMP5.daily$WT29 <- final.model2[1, 29 ]+final.model2[2, 29 ]*CIMP5.daily$JulianDate.1 + Air.Val.Coef.1[1,1]*Ratio.Coef1[1,1]*sin(2*pi*CIMP5.daily$DOY/365*1)+Air.Val.Coef.1[1,2]*Ratio.Coef1[1,2]*cos(2*pi*CIMP5.daily$DOY/365*1)

CIMP5.daily$WT30 <- final.model2[1, 30 ]+final.model2[2, 30]*CIMP5.daily$JulianDate.1 + Air.Val.Coef.1[1,1]*Ratio.Coef1[1,1]*sin(2*pi*CIMP5.daily$DOY/365*1)+Air.Val.Coef.1[1,2]*Ratio.Coef1[1,2]*cos(2*pi*CIMP5.daily$DOY/365*1)

CIMP5.daily$WT31 <- final.model2[1, 31]+final.model2[2, 31]*CIMP5.daily$JulianDate.1 + Air.Val.Coef.1[1,1]*Ratio.Coef1[1,1]*sin(2*pi*CIMP5.daily$DOY/365*1)+Air.Val.Coef.1[1,2]*Ratio.Coef1[1,2]*cos(2*pi*CIMP5.daily$DOY/365*1)

CIMP5.daily$WT32 <- final.model2[1, 32]+final.model2[2, 32 ]*CIMP5.daily$JulianDate.1 + Air.Val.Coef.1[1,1]*Ratio.Coef1[1,1]*sin(2*pi*CIMP5.daily$DOY/365*1)+Air.Val.Coef.1[1,2]*Ratio.Coef1[1,2]*cos(2*pi*CIMP5.daily$DOY/365*1)

CIMP5.daily$WT33 <- final.model2[1, 33]+final.model2[2, 33]*CIMP5.daily$JulianDate.1 + Air.Val.Coef.1[1,1]*Ratio.Coef1[1,1]*sin(2*pi*CIMP5.daily$DOY/365*1)+Air.Val.Coef.1[1,2]*Ratio.Coef1[1,2]*cos(2*pi*CIMP5.daily$DOY/365*1)

CIMP5.daily$WT34 <- final.model2[1, 34]+final.model2[2, 34]*CIMP5.daily$JulianDate.1 + Air.Val.Coef.1[1,1]*Ratio.Coef1[1,1]*sin(2*pi*CIMP5.daily$DOY/365*1)+Air.Val.Coef.1[1,2]*Ratio.Coef1[1,2]*cos(2*pi*CIMP5.daily$DOY/365*1)

CIMP5.daily$WT35 <- final.model2[1, 35]+final.model2[2, 35]*CIMP5.daily$JulianDate.1 + Air.Val.Coef.1[1,1]*Ratio.Coef1[1,1]*sin(2*pi*CIMP5.daily$DOY/365*1)+Air.Val.Coef.1[1,2]*Ratio.Coef1[1,2]*cos(2*pi*CIMP5.daily$DOY/365*1)

CIMP5.daily$WT36 <- final.model2[1, 36]+final.model2[2, 36]*CIMP5.daily$JulianDate.1 + Air.Val.Coef.1[1,1]*Ratio.Coef1[1,1]*sin(2*pi*CIMP5.daily$DOY/365*1)+Air.Val.Coef.1[1,2]*Ratio.Coef1[1,2]*cos(2*pi*CIMP5.daily$DOY/365*1)

CIMP5.daily$WT37 <- final.model2[1, 37]+final.model2[2, 37]*CIMP5.daily$JulianDate.1 + Air.Val.Coef.1[1,1]*Ratio.Coef1[1,1]*sin(2*pi*CIMP5.daily$DOY/365*1)+Air.Val.Coef.1[1,2]*Ratio.Coef1[1,2]*cos(2*pi*CIMP5.daily$DOY/365*1)

CIMP5.daily$WT38 <- final.model2[1, 38]+final.model2[2, 38]*CIMP5.daily$JulianDate.1 + Air.Val.Coef.1[1,1]*Ratio.Coef1[1,1]*sin(2*pi*CIMP5.daily$DOY/365*1)+Air.Val.Coef.1[1,2]*Ratio.Coef1[1,2]*cos(2*pi*CIMP5.daily$DOY/365*1)

CIMP5.daily$WT39 <- final.model2[1, 39]+final.model2[2, 39]*CIMP5.daily$JulianDate.1 + Air.Val.Coef.1[1,1]*Ratio.Coef1[1,1]*sin(2*pi*CIMP5.daily$DOY/365*1)+Air.Val.Coef.1[1,2]*Ratio.Coef1[1,2]*cos(2*pi*CIMP5.daily$DOY/365*1)

CIMP5.daily$WT40 <- final.model2[1, 40]+final.model2[2, 40]*CIMP5.daily$JulianDate.1 + Air.Val.Coef.1[1,1]*Ratio.Coef1[1,1]*sin(2*pi*CIMP5.daily$DOY/365*1)+Air.Val.Coef.1[1,2]*Ratio.Coef1[1,2]*cos(2*pi*CIMP5.daily$DOY/365*1)

CIMP5.daily$WT41 <- final.model2[1, 41]+final.model2[2, 41]*CIMP5.daily$JulianDate.1 + Air.Val.Coef.1[1,1]*Ratio.Coef1[1,1]*sin(2*pi*CIMP5.daily$DOY/365*1)+Air.Val.Coef.1[1,2]*Ratio.Coef1[1,2]*cos(2*pi*CIMP5.daily$DOY/365*1)

CIMP5.daily$WTmean <- rowMeans(CIMP5.daily[,53:93])

#make a matrix of only water temp prediction data for sd calculations

library(matrixStats)

wt.matrix <- data.matrix(CIMP5.daily[,53:93],rownames.force = NA)

CIMP5.daily$WTsd <- rowSds(wt.matrix)

CIMP5.daily$WTmin <- rowMins(wt.matrix)

CIMP5.daily$WTmax <- rowMaxs(wt.matrix)

#create subset of predicted CIMP5 water temp for climatology

CIMP5.daily2 <- subset(CIMP5.daily, Year>2069)

WT.climatology.mean<- tapply(CIMP5.daily2$WTmean, CIMP5.daily2$DOY, mean, na.rm=TRUE)

WT.climatology.min <- tapply(CIMP5.daily2$WTmin, CIMP5.daily2$DOY, mean, na.rm=TRUE)

WT.climatology.max <- tapply(CIMP5.daily2$WTmax, CIMP5.daily2$DOY, mean, na.rm=TRUE)

WT.climatology.sd <- tapply(CIMP5.daily2$WTsd, CIMP5.daily2$DOY, mean, na.rm=TRUE)

WT.climatology <- as.data.frame(WT.climatology.mean)

colnames(WT.climatology) <- "mean"

WT.climatology$min <- WT.climatology.min

WT.climatology$max <- WT.climatology.max

WT.climatology$DOY <- seq(1,366,1)

WT.climatology$SD <- WT.climatology.sd

WT.climatology$SD.1 <- WT.climatology$mean+WT.climatology.sd

WT.climatology$SD.2 <- WT.climatology$mean-WT.climatology.sd

#####################################################################

#winter length and survival

#count number of observations per year in historical data

pier.obs <- aggregate(cbind(count = CombinedWaterTemp) ~ Year,

data = pierdata,

FUN = function(x){NROW(x)})

#subset pier data so it is just winter

pierdata.winter <- subset(pierdata,pierdata$CombinedWaterTemp<9)

#count number of observations that are in winter per year

winter.obs <- aggregate(cbind(count = CombinedWaterTemp) ~ Year,

data = pierdata.winter,

FUN = function(x){NROW(x)})

pier.obs$winter <- winter.obs$count+5

pier.obs$percent <- (pier.obs$winter/pier.obs$count)*100

#subset extended data so it is just winter

pierdata.extend.winter1 <- subset(pierdata.extend,pierdata.extend$mean<9)

#count number of observations that are in winter per year

winter.obs.extend1 <- aggregate(cbind(count = mean) ~ year,

data = pierdata.extend.winter1,

FUN = function(x){NROW(x)})

winter.obs.extend1$percent <- (winter.obs.extend1$count/365)*100

extend.obs <- seq(2017,2099,1)

extend.obs <- as.data.frame(extend.obs)

extend.obs$count <- 365

extend.obs$winter <- winter.obs.extend1$count

extend.obs$percent <- winter.obs.extend1$percent

colnames(extend.obs) <- c("Year","count","winter","percent")

#subset CIMP5 daily data so it is just winter

CIMP5.daily.winter <- subset(CIMP5.daily,CIMP5.daily$WTmean<9)

#count number of observations that are in winter per year

CIMP5.winter.obs.extend1 <- aggregate(cbind(count = WTmean) ~ Year,

data = CIMP5.daily.winter,

FUN = function(x){NROW(x)})

CIMP5.winter.obs.extend1$percent <- (CIMP5.winter.obs.extend1$count/365)*100

CIMP5.winter.obs.extend1 <- subset(CIMP5.winter.obs.extend1,Year>2016)

extend.obs$CIMP5.count <- CIMP5.winter.obs.extend1$count

extend.obs$CIMP5.percent <- CIMP5.winter.obs.extend1$percent

##############################################

#get estimates and error bands for each model separately

#subset data so it is just winter

CIMP5.daily2 <- CIMP5.daily[,53:93]

CIMP5.daily2$Year <- CIMP5.daily$Year

CIMP5.daily2$Year2 <- CIMP5.daily$Year2

winter.by.year <- sapply(split(CIMP5.daily2, CIMP5.daily2$Year), function(x) apply(x,2, function(x) length(x[x<9])))

winter.by.year <- as.data.frame(winter.by.year)

winter.by.year <- t(winter.by.year)

winter.by.year <- as.data.frame(winter.by.year)

winter.by.year$Year <- seq(1950,2099,1)

winter.by.year$mean <- rowMeans(winter.by.year[,1:41])

library(matrixStats)

wt.matrix2 <- data.matrix(winter.by.year[,1:41],rownames.force = NA)

winter.by.year$SD <- rowSds(wt.matrix2)

winter.by.year$Min <- rowMins(wt.matrix2)

winter.by.year$Max <- rowMaxs(wt.matrix2)

winter.by.year$SD1 <- winter.by.year$mean+winter.by.year$SD

winter.by.year$SD2 <- winter.by.year$mean-winter.by.year$SD

winter.by.year2 <- subset(winter.by.year,Year>1950)

#######################################################

#survival estimates

#calculate mean winter temp by year from historical data

library(dplyr)

hist.mean <- tapply(pierdata.winter$CombinedWaterTemp, pierdata.winter$Year, mean, na.rm=TRUE)

historical.data <- as.data.frame(hist.mean)

colnames(historical.data) <- "meantemp"

historical.data$Year <- winter.obs$Year

#historical.data <- subset(historical.data,historical.data$Year<2017)

historical.data$length <- pier.obs$winter

historical.data$count <- pier.obs$count

#use equation from Bauer paper to calculate historical winter survival

#use mean salinity and crab size (mm) from 2015 experiment

#sal = 12, CW = 40

lamda=1/0.45

sal=12

size=40

historical.data$survival <- exp(-lamda*((historical.data$length)^lamda)*exp(-lamda*(3.59+(0.1*historical.data$meantemp)+

0.02*(sal)+0.03*(size))))

#get winter temp for each iteration of the extended trend

winter.by.year.extend.meantemp <- sapply(split(pierdata.extend2, pierdata.extend2$Year),

function(x) apply(x,2, function(x) mean(x[x<9])))

winter.by.year.extend.meantemp <- as.data.frame(winter.by.year.extend.meantemp)

winter.by.year.extend.meantemp <- t(winter.by.year.extend.meantemp)

winter.by.year.extend.meantemp <- as.data.frame(winter.by.year.extend.meantemp)

winter.by.year.extend.meantemp$Year <- seq(2017,2099,1)

#make empty matrix for survival for each model iteration

extended.survival.matrix=matrix(nrow=83,ncol=50)

extended.survival.matrix[,1] <- exp(-lamda*((winter.by.year.extend$WT1.1)^lamda)*

exp(-lamda*(3.59+(0.1*winter.by.year.extend.meantemp$WT1.1)+

0.02*(sal)+0.03*(size))))

extended.survival.matrix[,2] <- exp(-lamda*((winter.by.year.extend$WT1.2)^lamda)*

exp(-lamda*(3.59+(0.1*winter.by.year.extend.meantemp$WT1.2)+

0.02*(sal)+0.03*(size))))

extended.survival.matrix[,3] <- exp(-lamda*((winter.by.year.extend$WT1.3)^lamda)*

exp(-lamda*(3.59+(0.1*winter.by.year.extend.meantemp$WT1.3)+

0.02*(sal)+0.03*(size))))

extended.survival.matrix[,4] <- exp(-lamda*((winter.by.year.extend$WT1.4)^lamda)*

exp(-lamda*(3.59+(0.1*winter.by.year.extend.meantemp$WT1.4)+

0.02*(sal)+0.03*(size))))

extended.survival.matrix[,5] <- exp(-lamda*((winter.by.year.extend$WT1.5)^lamda)*

exp(-lamda*(3.59+(0.1*winter.by.year.extend.meantemp$WT1.5)+

0.02*(sal)+0.03*(size))))

extended.survival.matrix[,6] <- exp(-lamda*((winter.by.year.extend$WT1.6)^lamda)*

exp(-lamda*(3.59+(0.1*winter.by.year.extend.meantemp$WT1.6)+

0.02*(sal)+0.03*(size))))

extended.survival.matrix[,7] <- exp(-lamda*((winter.by.year.extend$WT1.7)^lamda)*

exp(-lamda*(3.59+(0.1*winter.by.year.extend.meantemp$WT1.7)+

0.02*(sal)+0.03*(size))))

extended.survival.matrix[,8] <- exp(-lamda*((winter.by.year.extend$WT1.8)^lamda)*

exp(-lamda*(3.59+(0.1*winter.by.year.extend.meantemp$WT1.8)+

0.02*(sal)+0.03*(size))))

extended.survival.matrix[,9] <- exp(-lamda*((winter.by.year.extend$WT1.9)^lamda)*

exp(-lamda*(3.59+(0.1*winter.by.year.extend.meantemp$WT1.9)+

0.02*(sal)+0.03*(size))))

extended.survival.matrix[,10] <- exp(-lamda*((winter.by.year.extend$WT1.10)^lamda)*

exp(-lamda*(3.59+(0.1*winter.by.year.extend.meantemp$WT1.10)+

0.02*(sal)+0.03*(size))))

extended.survival.matrix[,11] <- exp(-lamda*((winter.by.year.extend$WT1.11)^lamda)*

exp(-lamda*(3.59+(0.1*winter.by.year.extend.meantemp$WT1.11)+

0.02*(sal)+0.03*(size))))

extended.survival.matrix[,12] <- exp(-lamda*((winter.by.year.extend$WT1.12)^lamda)*

exp(-lamda*(3.59+(0.1*winter.by.year.extend.meantemp$WT1.12)+

0.02*(sal)+0.03*(size))))

extended.survival.matrix[,13] <- exp(-lamda*((winter.by.year.extend$WT1.13)^lamda)*

exp(-lamda*(3.59+(0.1*winter.by.year.extend.meantemp$WT1.13)+

0.02*(sal)+0.03*(size))))

extended.survival.matrix[,14] <- exp(-lamda*((winter.by.year.extend$WT1.14)^lamda)*

exp(-lamda*(3.59+(0.1*winter.by.year.extend.meantemp$WT1.14)+

0.02*(sal)+0.03*(size))))

extended.survival.matrix[,15] <- exp(-lamda*((winter.by.year.extend$WT1.15)^lamda)*

exp(-lamda*(3.59+(0.1*winter.by.year.extend.meantemp$WT1.15)+

0.02*(sal)+0.03*(size))))

extended.survival.matrix[,16] <- exp(-lamda*((winter.by.year.extend$WT1.16)^lamda)*

exp(-lamda*(3.59+(0.1*winter.by.year.extend.meantemp$WT1.16)+

0.02*(sal)+0.03*(size))))

extended.survival.matrix[,17] <- exp(-lamda*((winter.by.year.extend$WT1.17)^lamda)*

exp(-lamda*(3.59+(0.1*winter.by.year.extend.meantemp$WT1.17)+

0.02*(sal)+0.03*(size))))

extended.survival.matrix[,18] <- exp(-lamda*((winter.by.year.extend$WT1.18)^lamda)*

exp(-lamda*(3.59+(0.1*winter.by.year.extend.meantemp$WT1.18)+

0.02*(sal)+0.03*(size))))

extended.survival.matrix[,19] <- exp(-lamda*((winter.by.year.extend$WT1.19)^lamda)*

exp(-lamda*(3.59+(0.1*winter.by.year.extend.meantemp$WT1.19)+

0.02*(sal)+0.03*(size))))

extended.survival.matrix[,20] <- exp(-lamda*((winter.by.year.extend$WT1.20)^lamda)*

exp(-lamda*(3.59+(0.1*winter.by.year.extend.meantemp$WT1.20)+

0.02*(sal)+0.03*(size))))

extended.survival.matrix[,21] <- exp(-lamda*((winter.by.year.extend$WT1.21)^lamda)*

exp(-lamda*(3.59+(0.1*winter.by.year.extend.meantemp$WT1.21)+

0.02*(sal)+0.03*(size))))

extended.survival.matrix[,22] <- exp(-lamda*((winter.by.year.extend$WT1.22)^lamda)*

exp(-lamda*(3.59+(0.1*winter.by.year.extend.meantemp$WT1.22)+

0.02*(sal)+0.03*(size))))

extended.survival.matrix[,23] <- exp(-lamda*((winter.by.year.extend$WT1.23)^lamda)*

exp(-lamda*(3.59+(0.1*winter.by.year.extend.meantemp$WT1.23)+

0.02*(sal)+0.03*(size))))

extended.survival.matrix[,24] <- exp(-lamda*((winter.by.year.extend$WT1.24)^lamda)*

exp(-lamda*(3.59+(0.1*winter.by.year.extend.meantemp$WT1.24)+

0.02*(sal)+0.03*(size))))

extended.survival.matrix[,25] <- exp(-lamda*((winter.by.year.extend$WT1.25)^lamda)*

exp(-lamda*(3.59+(0.1*winter.by.year.extend.meantemp$WT1.25)+

0.02*(sal)+0.03*(size))))

extended.survival.matrix[,26] <- exp(-lamda*((winter.by.year.extend$WT1.26)^lamda)*

exp(-lamda*(3.59+(0.1*winter.by.year.extend.meantemp$WT1.26)+

0.02*(sal)+0.03*(size))))

extended.survival.matrix[,27] <- exp(-lamda*((winter.by.year.extend$WT1.27)^lamda)*

exp(-lamda*(3.59+(0.1*winter.by.year.extend.meantemp$WT1.27)+

0.02*(sal)+0.03*(size))))

extended.survival.matrix[,28] <- exp(-lamda*((winter.by.year.extend$WT1.28)^lamda)*

exp(-lamda*(3.59+(0.1*winter.by.year.extend.meantemp$WT1.28)+

0.02*(sal)+0.03*(size))))

extended.survival.matrix[,29] <- exp(-lamda*((winter.by.year.extend$WT1.29)^lamda)*

exp(-lamda*(3.59+(0.1*winter.by.year.extend.meantemp$WT1.29)+

0.02*(sal)+0.03*(size))))

extended.survival.matrix[,30] <- exp(-lamda*((winter.by.year.extend$WT1.30)^lamda)*

exp(-lamda*(3.59+(0.1*winter.by.year.extend.meantemp$WT1.30)+

0.02*(sal)+0.03*(size))))

extended.survival.matrix[,31] <- exp(-lamda*((winter.by.year.extend$WT1.31)^lamda)*

exp(-lamda*(3.59+(0.1*winter.by.year.extend.meantemp$WT1.31)+

0.02*(sal)+0.03*(size))))

extended.survival.matrix[,32] <- exp(-lamda*((winter.by.year.extend$WT1.32)^lamda)*

exp(-lamda*(3.59+(0.1*winter.by.year.extend.meantemp$WT1.32)+

0.02*(sal)+0.03*(size))))

extended.survival.matrix[,33] <- exp(-lamda*((winter.by.year.extend$WT1.33)^lamda)*

exp(-lamda*(3.59+(0.1*winter.by.year.extend.meantemp$WT1.33)+

0.02*(sal)+0.03*(size))))

extended.survival.matrix[,34] <- exp(-lamda*((winter.by.year.extend$WT1.34)^lamda)*

exp(-lamda*(3.59+(0.1*winter.by.year.extend.meantemp$WT1.34)+

0.02*(sal)+0.03*(size))))

extended.survival.matrix[,35] <- exp(-lamda*((winter.by.year.extend$WT1.35)^lamda)*

exp(-lamda*(3.59+(0.1*winter.by.year.extend.meantemp$WT1.35)+

0.02*(sal)+0.03*(size))))

extended.survival.matrix[,36] <- exp(-lamda*((winter.by.year.extend$WT1.36)^lamda)*

exp(-lamda*(3.59+(0.1*winter.by.year.extend.meantemp$WT1.36)+

0.02*(sal)+0.03*(size))))

extended.survival.matrix[,37] <- exp(-lamda*((winter.by.year.extend$WT1.37)^lamda)*

exp(-lamda*(3.59+(0.1*winter.by.year.extend.meantemp$WT1.37)+

0.02*(sal)+0.03*(size))))

extended.survival.matrix[,38] <- exp(-lamda*((winter.by.year.extend$WT1.38)^lamda)*

exp(-lamda*(3.59+(0.1*winter.by.year.extend.meantemp$WT1.38)+

0.02*(sal)+0.03*(size))))

extended.survival.matrix[,39] <- exp(-lamda*((winter.by.year.extend$WT1.39)^lamda)*

exp(-lamda*(3.59+(0.1*winter.by.year.extend.meantemp$WT1.39)+

0.02*(sal)+0.03*(size))))

extended.survival.matrix[,40] <- exp(-lamda*((winter.by.year.extend$WT1.40)^lamda)*

exp(-lamda*(3.59+(0.1*winter.by.year.extend.meantemp$WT1.40)+

0.02*(sal)+0.03*(size))))

extended.survival.matrix[,41] <- exp(-lamda*((winter.by.year.extend$WT1.41)^lamda)*

exp(-lamda*(3.59+(0.1*winter.by.year.extend.meantemp$WT1.41)+

0.02*(sal)+0.03*(size))))

extended.survival.matrix[,42] <- exp(-lamda*((winter.by.year.extend$WT1.42)^lamda)*

exp(-lamda*(3.59+(0.1*winter.by.year.extend.meantemp$WT1.42)+

0.02*(sal)+0.03*(size))))

extended.survival.matrix[,43] <- exp(-lamda*((winter.by.year.extend$WT1.43)^lamda)*

exp(-lamda*(3.59+(0.1*winter.by.year.extend.meantemp$WT1.43)+

0.02*(sal)+0.03*(size))))

extended.survival.matrix[,44] <- exp(-lamda*((winter.by.year.extend$WT1.44)^lamda)*

exp(-lamda*(3.59+(0.1*winter.by.year.extend.meantemp$WT1.44)+

0.02*(sal)+0.03*(size))))

extended.survival.matrix[,45] <- exp(-lamda*((winter.by.year.extend$WT1.45)^lamda)*

exp(-lamda*(3.59+(0.1*winter.by.year.extend.meantemp$WT1.45)+

0.02*(sal)+0.03*(size))))

extended.survival.matrix[,46] <- exp(-lamda*((winter.by.year.extend$WT1.46)^lamda)*

exp(-lamda*(3.59+(0.1*winter.by.year.extend.meantemp$WT1.46)+

0.02*(sal)+0.03*(size))))

extended.survival.matrix[,47] <- exp(-lamda*((winter.by.year.extend$WT1.47)^lamda)*

exp(-lamda*(3.59+(0.1*winter.by.year.extend.meantemp$WT1.47)+

0.02*(sal)+0.03*(size))))

extended.survival.matrix[,48] <- exp(-lamda*((winter.by.year.extend$WT1.48)^lamda)*

exp(-lamda*(3.59+(0.1*winter.by.year.extend.meantemp$WT1.48)+

0.02*(sal)+0.03*(size))))

extended.survival.matrix[,49] <- exp(-lamda*((winter.by.year.extend$WT1.49)^lamda)*

exp(-lamda*(3.59+(0.1*winter.by.year.extend.meantemp$WT1.49)+

0.02*(sal)+0.03*(size))))

extended.survival.matrix[,50] <- exp(-lamda*((winter.by.year.extend$WT1.50)^lamda)*

exp(-lamda*(3.59+(0.1*winter.by.year.extend.meantemp$WT1.50)+

0.02*(sal)+0.03*(size))))

#make a new data frame for mean, sd of survival from all model iterations

extended.survival=matrix(nrow=83,ncol=1)

extended.survival <- as.data.frame(extended.survival)

#calculate sd, min, max on matrix

library(matrixStats)

extended.survival[,1] <- seq(2017,2099,1)

colnames(extended.survival) <- "Year"

extended.survival$SD <- rowSds(extended.survival.matrix)

extended.survival$Min <- rowMins(extended.survival.matrix)

extended.survival$Max <- rowMaxs(extended.survival.matrix)

#make matrix a data frame

extended.survival.matrix <- as.data.frame(extended.survival.matrix)

#calculate mean and sd bands

extended.survival$survival<- rowMeans(extended.survival.matrix[,1:50])

extended.survival$SD1 <- extended.survival$survival+extended.survival$SD

extended.survival$SD2 <- extended.survival$survival-extended.survival$SD

#get mean temp during winter of each year for each CMIP5 model

winter.by.year.meantemp <- sapply(split(CIMP5.daily2, CIMP5.daily2$Year),

function(x) apply(x,2, function(x) mean(x[x<9])))

winter.by.year.meantemp <- as.data.frame(winter.by.year.meantemp)

winter.by.year.meantemp <- t(winter.by.year.meantemp)

winter.by.year.meantemp <- as.data.frame(winter.by.year.meantemp)

winter.by.year.meantemp$Year <- seq(1950,2099,1)

#make empty matrix for survival for each model iteration

CMIP.survival.matrix=matrix(nrow=150,ncol=41)

CMIP.survival.matrix[,1] <- exp(-lamda*((winter.by.year$WT1)^lamda)*

exp(-lamda*(3.59+(0.1*winter.by.year.meantemp$WT1)+

0.02*(sal)+0.03*(size))))

CMIP.survival.matrix[,2] <- exp(-lamda*((winter.by.year$WT2)^lamda)*

exp(-lamda*(3.59+(0.1*winter.by.year.meantemp$WT2)+

0.02*(sal)+0.03*(size))))

CMIP.survival.matrix[,3] <- exp(-lamda*((winter.by.year$WT3)^lamda)*

exp(-lamda*(3.59+(0.1*winter.by.year.meantemp$WT3)+

0.02*(sal)+0.03*(size))))

CMIP.survival.matrix[,4] <- exp(-lamda*((winter.by.year$WT4)^lamda)*

exp(-lamda*(3.59+(0.1*winter.by.year.meantemp$WT4)+

0.02*(sal)+0.03*(size))))

CMIP.survival.matrix[,5] <- exp(-lamda*((winter.by.year$WT5)^lamda)*

exp(-lamda*(3.59+(0.1*winter.by.year.meantemp$WT5)+

0.02*(sal)+0.03*(size))))

CMIP.survival.matrix[,6] <- exp(-lamda*((winter.by.year$WT6)^lamda)*

exp(-lamda*(3.59+(0.1*winter.by.year.meantemp$WT6)+

0.02*(sal)+0.03*(size))))

CMIP.survival.matrix[,7] <- exp(-lamda*((winter.by.year$WT7)^lamda)*

exp(-lamda*(3.59+(0.1*winter.by.year.meantemp$WT7)+

0.02*(sal)+0.03*(size))))

CMIP.survival.matrix[,8] <- exp(-lamda*((winter.by.year$WT8)^lamda)*

exp(-lamda*(3.59+(0.1*winter.by.year.meantemp$WT8)+

0.02*(sal)+0.03*(size))))

CMIP.survival.matrix[,9] <- exp(-lamda*((winter.by.year$WT9)^lamda)*

exp(-lamda*(3.59+(0.1*winter.by.year.meantemp$WT9)+

0.02*(sal)+0.03*(size))))

CMIP.survival.matrix[,10] <- exp(-lamda*((winter.by.year$WT10)^lamda)*

exp(-lamda*(3.59+(0.1*winter.by.year.meantemp$WT10)+

0.02*(sal)+0.03*(size))))

CMIP.survival.matrix[,11] <- exp(-lamda*((winter.by.year$WT11)^lamda)*

exp(-lamda*(3.59+(0.1*winter.by.year.meantemp$WT11)+

0.02*(sal)+0.03*(size))))

CMIP.survival.matrix[,12] <- exp(-lamda*((winter.by.year$WT12)^lamda)*

exp(-lamda*(3.59+(0.1*winter.by.year.meantemp$WT12)+

0.02*(sal)+0.03*(size))))

CMIP.survival.matrix[,13] <- exp(-lamda*((winter.by.year$WT13)^lamda)*

exp(-lamda*(3.59+(0.1*winter.by.year.meantemp$WT13)+

0.02*(sal)+0.03*(size))))

CMIP.survival.matrix[,14] <- exp(-lamda*((winter.by.year$WT14)^lamda)*

exp(-lamda*(3.59+(0.1*winter.by.year.meantemp$WT14)+

0.02*(sal)+0.03*(size))))

CMIP.survival.matrix[,15] <- exp(-lamda*((winter.by.year$WT15)^lamda)*

exp(-lamda*(3.59+(0.1*winter.by.year.meantemp$WT15)+

0.02*(sal)+0.03*(size))))

CMIP.survival.matrix[,16] <- exp(-lamda*((winter.by.year$WT16)^lamda)*

exp(-lamda*(3.59+(0.1*winter.by.year.meantemp$WT16)+

0.02*(sal)+0.03*(size))))

CMIP.survival.matrix[,17] <- exp(-lamda*((winter.by.year$WT17)^lamda)*

exp(-lamda*(3.59+(0.1*winter.by.year.meantemp$WT17)+

0.02*(sal)+0.03*(size))))

CMIP.survival.matrix[,18] <- exp(-lamda*((winter.by.year$WT18)^lamda)*

exp(-lamda*(3.59+(0.1*winter.by.year.meantemp$WT18)+

0.02*(sal)+0.03*(size))))

CMIP.survival.matrix[,19] <- exp(-lamda*((winter.by.year$WT19)^lamda)*

exp(-lamda*(3.59+(0.1*winter.by.year.meantemp$WT19)+

0.02*(sal)+0.03*(size))))

CMIP.survival.matrix[,20] <- exp(-lamda*((winter.by.year$WT20)^lamda)*

exp(-lamda*(3.59+(0.1*winter.by.year.meantemp$WT20)+

0.02*(sal)+0.03*(size))))

CMIP.survival.matrix[,21] <- exp(-lamda*((winter.by.year$WT21)^lamda)*

exp(-lamda*(3.59+(0.1*winter.by.year.meantemp$WT21)+

0.02*(sal)+0.03*(size))))

CMIP.survival.matrix[,22] <- exp(-lamda*((winter.by.year$WT22)^lamda)*

exp(-lamda*(3.59+(0.1*winter.by.year.meantemp$WT22)+

0.02*(sal)+0.03*(size))))

CMIP.survival.matrix[,23] <- exp(-lamda*((winter.by.year$WT23)^lamda)*

exp(-lamda*(3.59+(0.1*winter.by.year.meantemp$WT23)+

0.02*(sal)+0.03*(size))))

CMIP.survival.matrix[,24] <- exp(-lamda*((winter.by.year$WT24)^lamda)*

exp(-lamda*(3.59+(0.1*winter.by.year.meantemp$WT24)+

0.02*(sal)+0.03*(size))))

CMIP.survival.matrix[,25] <- exp(-lamda*((winter.by.year$WT25)^lamda)*

exp(-lamda*(3.59+(0.1*winter.by.year.meantemp$WT25)+

0.02*(sal)+0.03*(size))))

CMIP.survival.matrix[,26] <- exp(-lamda*((winter.by.year$WT26)^lamda)*

exp(-lamda*(3.59+(0.1*winter.by.year.meantemp$WT26)+

0.02*(sal)+0.03*(size))))

CMIP.survival.matrix[,27] <- exp(-lamda*((winter.by.year$WT27)^lamda)*

exp(-lamda*(3.59+(0.1*winter.by.year.meantemp$WT27)+

0.02*(sal)+0.03*(size))))

CMIP.survival.matrix[,28] <- exp(-lamda*((winter.by.year$WT28)^lamda)*

exp(-lamda*(3.59+(0.1*winter.by.year.meantemp$WT28)+

0.02*(sal)+0.03*(size))))

CMIP.survival.matrix[,29] <- exp(-lamda*((winter.by.year$WT29)^lamda)*

exp(-lamda*(3.59+(0.1*winter.by.year.meantemp$WT29)+

0.02*(sal)+0.03*(size))))

CMIP.survival.matrix[,30] <- exp(-lamda*((winter.by.year$WT30)^lamda)*

exp(-lamda*(3.59+(0.1*winter.by.year.meantemp$WT30)+

0.02*(sal)+0.03*(size))))

CMIP.survival.matrix[,31] <- exp(-lamda*((winter.by.year$WT31)^lamda)*

exp(-lamda*(3.59+(0.1*winter.by.year.meantemp$WT31)+

0.02*(sal)+0.03*(size))))

CMIP.survival.matrix[,32] <- exp(-lamda*((winter.by.year$WT32)^lamda)*

exp(-lamda*(3.59+(0.1*winter.by.year.meantemp$WT32)+

0.02*(sal)+0.03*(size))))

CMIP.survival.matrix[,33] <- exp(-lamda*((winter.by.year$WT33)^lamda)*

exp(-lamda*(3.59+(0.1*winter.by.year.meantemp$WT33)+

0.02*(sal)+0.03*(size))))

CMIP.survival.matrix[,34] <- exp(-lamda*((winter.by.year$WT34)^lamda)*

exp(-lamda*(3.59+(0.1*winter.by.year.meantemp$WT34)+

0.02*(sal)+0.03*(size))))

CMIP.survival.matrix[,35] <- exp(-lamda*((winter.by.year$WT35)^lamda)*

exp(-lamda*(3.59+(0.1*winter.by.year.meantemp$WT35)+

0.02*(sal)+0.03*(size))))

CMIP.survival.matrix[,36] <- exp(-lamda*((winter.by.year$WT36)^lamda)*

exp(-lamda*(3.59+(0.1*winter.by.year.meantemp$WT36)+

0.02*(sal)+0.03*(size))))

CMIP.survival.matrix[,37] <- exp(-lamda*((winter.by.year$WT37)^lamda)*

exp(-lamda*(3.59+(0.1*winter.by.year.meantemp$WT37)+

0.02*(sal)+0.03*(size))))

CMIP.survival.matrix[,38] <- exp(-lamda*((winter.by.year$WT38)^lamda)*

exp(-lamda*(3.59+(0.1*winter.by.year.meantemp$WT38)+

0.02*(sal)+0.03*(size))))

CMIP.survival.matrix[,39] <- exp(-lamda*((winter.by.year$WT39)^lamda)*

exp(-lamda*(3.59+(0.1*winter.by.year.meantemp$WT39)+

0.02*(sal)+0.03*(size))))

CMIP.survival.matrix[,40] <- exp(-lamda*((winter.by.year$WT40)^lamda)*

exp(-lamda*(3.59+(0.1*winter.by.year.meantemp$WT40)+

0.02*(sal)+0.03*(size))))

CMIP.survival.matrix[,41] <- exp(-lamda*((winter.by.year$WT41)^lamda)*

exp(-lamda*(3.59+(0.1*winter.by.year.meantemp$WT41)+

0.02*(sal)+0.03*(size))))

#make a new data frame for mean, sd of survival from all model iterations

CMIP.survival=matrix(nrow=150,ncol=1)

CMIP.survival <- as.data.frame(CMIP.survival)

#calculate sd, min, max on matrix

library(matrixStats)

CMIP.survival[,1] <- seq(1950,2099,1)

colnames(CMIP.survival) <- "Year"

CMIP.survival$SD <- rowSds(CMIP.survival.matrix)

CMIP.survival$Min <- rowMins(CMIP.survival.matrix)

CMIP.survival$Max <- rowMaxs(CMIP.survival.matrix)

#make matrix a data frame

CMIP.survival.matrix <- as.data.frame(CMIP.survival.matrix)

#calculate mean and sd bands

CMIP.survival$survival<- rowMeans(CMIP.survival.matrix[,1:41])

CMIP.survival$SD1 <- CMIP.survival$survival+CMIP.survival$SD

CMIP.survival$SD2 <- CMIP.survival$survival-CMIP.survival$SD
